# DIA-based proteomic insights into quality deterioration and spoilage biomarkers of chilled anchovies (*Stolephorus commersonii*)

**DOI:** 10.1016/j.fochx.2026.104730

**Published:** 2026-11-04

**Authors:** Shihao Chen, Jingfu Bai, Shixian Yin, Wenli Kang, Hu Hou, Xiayin Ma

**Affiliations:** a School of Food Science and Bioengineering, Changsha University of Science and Technology, 960, 2nd Section, Wanjiali South RD, Changsha 410114, China; b Jinzai Food Group Co., Ltd., South Baishiling Road, Kangwang Industrial Park Economic & Technological Development Zone, Yueyang, Hunan, China; c State Key Laboratory of Marine Food Processing & Safety Control, College of Food Science and Engineering, Ocean University of China, No. 1299, Sansha Road, Qingdao 266404, China; d Hunan Provincial Engineering Research Center for Healthy Snack Food, China

**Keywords:** Anchovy, Postmortem autolysis, Physiology and biochemistry, Histological changes, DIA proteomics, Biomarkers

## Abstract

Anchovy (*Stolephorus commersonii*) rapidly undergoes postmortem autolysis. This study investigated chilled anchovy degradation over 7 days at 4 °C using physicochemical, histological, and enzymatic analyses, coupled with DIA-based comparative proteomics between day 1 and day 7 samples. Days 1–3 provided optimal processing quality, whereas Days 4–5 marked a critical turning point: fish surface degraded, cathepsin B/L and catalase activities peaked, K-value exceeded 40%, and myofibrils collapsed with *Z*-disc disintegration. TVB-N crossed the freshness threshold (20 mg/100 g) at Days 5–6, though histamine stayed within safe limits. Comparative DIA proteomics between day 1 (fresh) and day 7 (spoiled) identified key proteins linked to myofibrillar collapse, mitochondrial apoptosis, energy failure, calcium storm, and oxidative stress. Promising freshness biomarkers, including type I keratin, hemoglobin subunits, HSP70, histone H1, FABP, and cytochrome *c* oxidase, were identified in day 7 samples relative to day 1.

## Highlights

• Days 1–3 post-harvest represent the golden processing window for anchovies.
• Days 4–5 mark the critical quality turning point driven by autolysis.
• DIA proteomics revealed calcium storm and mitochondrial apoptosis pathways.
• Key protein biomarkers were identified for objective freshness monitoring.

## Graphical abstract

**Figure:**
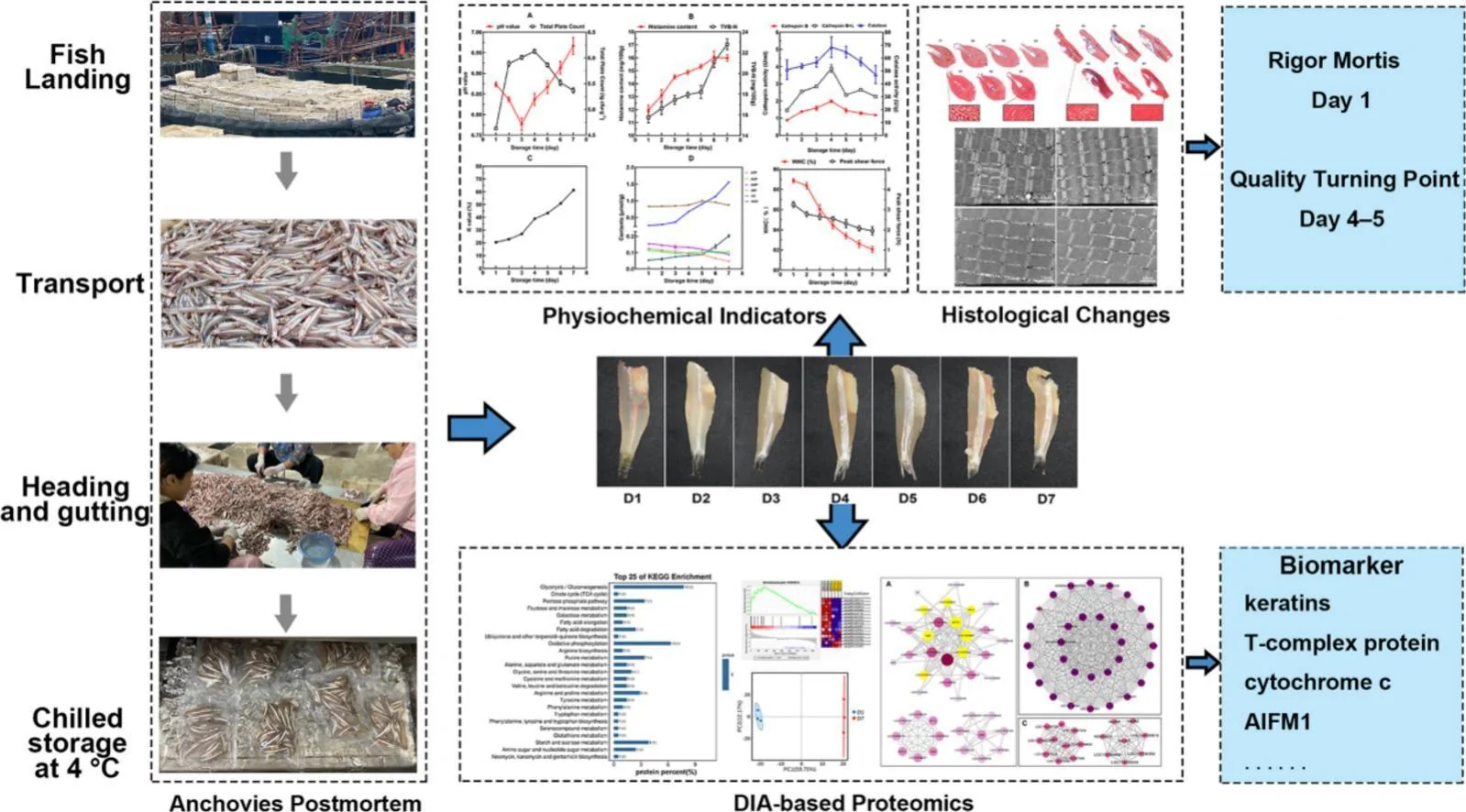

## 1 Introduction

Anchovy (*Stolephorus commersonii*) is a small, silvery pelagic fish widely distributed across the tropical and subtropical regions of the Indo-Pacific, including the coastal waters of China, India, and East Africa (FishSource, 2025). As a schooling, short-lived species, it plays a fundamental ecological role in marine food webs while maintaining high economic value and commercial landings (Maathuis et al., 2025). Anchovies exhibit high nutritional value, as they are rich in essential amino acids and unsaturated fatty acids such as DHA and EPA (Karslı et al., 2025). In Beihai, Guangxi, China, anchovies are harvested in large quantities and traditionally preserved in ice aboard fishing vessels. However, due to their small body size and active endogenous enzymes, anchovies are extremely perishable and suffer from rapid postmortem autolysis and quality deterioration (Huang et al., 2023; Mei et al., 2026). This rapid degradation poses significant logistical challenges for post-harvest handling, preservation, and downstream industrial utilization. Therefore, there is a critical need to evaluate post-harvest quality changes to support its application in processing.

Postmortem quality deterioration in fish is a complex physiological and biochemical process. Immediately following death, fish muscle undergoes marked sensory, structural, and physicochemical alterations, including muscle softening, loss of elasticity, surface browning, and the accumulation of off-odors (Li, Chang, et al., 2023). The underlying mechanisms involve rapid ATP depletion, mitochondrial structural decay, disruption of calcium homeostasis, and the continuous activation of endogenous proteases (Tie et al., 2022; Liu, Chen, et al., 2026). Under ischemic conditions, energy failure impairs energy-dependent calcium pumps, triggering an uncontrolled sarcoplasmic calcium surge. This calcium accumulation, coupled with mitochondrial collapse and postmortem acidification, strongly activates endogenous proteases to cleave key myofibrillar and cytoskeletal proteins, directly driving myofibrillar disassembly, reduced water-holding capacity, and textural softening (Hu et al., 2025). In order to elucidate these postmortem biochemical cascades, proteomics has been widely applied to map proteome profiles and discover biomarkers associated with fish postmortem (Hematyar et al., 2025). Xiang et al. (2022) analyzed sea bass fillets stored on ice via label-free proteomics, confirming that epigallocatechin gallate incorporated in agar oligosaccharide treatment suppressed calpain expression and delayed the degradation of structural proteins. Label-free proteomics was also used on pike eel during chilled storage, finding that cold stress caused severe depletion in membrane and cytoskeletal proteins (Yuan et al., 2022). In addition, 4D label-free proteomics was utilized to investigate grass carp fillets packaged with films, showing that active packaging preserved muscle stability by upregulating key structural proteins such as synaptopodin and Lumican (Zhang et al., 2025; Liu, Xie, et al., 2026). TMT-labeled quantitative proteomics was also used in hairtail muscles, demonstrating that higher storage temperatures accelerate the downregulation of structural proteins, such as myosin heavy chains (Yan et al., 2023). Furthermore, TMT-based quantitative phosphoproteomics was applied to analyze dynamic protein phosphorylation in structural proteins under refrigeration (Hu et al., 2026; Zhang et al., 2023). Compared with traditional data-dependent acquisition (DDA), data-independent acquisition (DIA) proteomics offers high throughput and high-precision proteomics by systematically fragmenting all precursor ions in sequential isolation windows, which provides comprehensive proteome coverage and minimal missing data (Zhang et al., 2026; Zou et al., 2025). In recent years, many studies used DIA-based proteomics, as Teng et al. (2024) identified isobutyryl-CoA dehydrogenase and staphylococcal nuclease domain-containing protein 1 as reliable biomarkers for monitoring textural deterioration of grass carp fillets.

In the present study, an integrated analytical framework combining sensory evaluation, physicochemical profiling, enzyme activity analysis, and tissue histology was used to evaluate postmortem quality deterioration in chilled anchovies stored at 4 °C over a 7-day period. The results indicated days 4–5 as the critical quality turning point for the optimal freshness window. DIA-based comparative proteomics showed that postmortem spoilage is driven by sarcoplasmic reticulum structural collapse and mitochondria-dependent apoptosis, and several proteins were selected as potential biomarkers based on their significant change in abundance.

## 2 Materials and methods

### 2.1 Raw anchovy sourcing and storage

Anchovies, with a body length of 5 to 8 cm, were harvested from the Beibu Gulf area close to Beihai and purchased at Beihai's Dianjian wharf. Upon arrival at the factory, the anchovies were manually beheaded and eviscerated within half a day of acquisition. Each 500 g portion was vacuum-sealed in a bag, quickly frozen with liquid nitrogen, and then transported to the laboratory via dry ice cold chain logistics. The sealed bags of anchovies were kept in an ice-covered refrigerator cabinet at 4 °C. Every eight hours, the melted ice was removed and replaced with fresh ice to maintain a consistently ice-chilled environment.

### 2.2 pH and total plate count determination

The pH of samples was determined with minor modifications based on Ding et al. (2024). Anchovies were rinsed with chilled distilled water, blotted dry, and coarsely chopped. After homogenization, 10 g of paste were taken and blended with 50 mL of CO_2_-free distilled water for 60 s on ice. After standing at room temperature for 30 min, the homogenate was filtered, and pH was measured using a benchtop pH meter.

Total plate count (TPC) was determined according to GB 4789.2-2022, 2022, with modifications for small fish. Edible anchovy muscle (1 g) was homogenized in sterile 0.85% NaCl (1:10, w/v) using a stomacher. Decimal serial dilutions were prepared to 10^−3^. For each dilution, 100 μL of the homogenate was spread-plated in duplicate on Plate Count Agar (PCA). Plates were incubated at 30 °C for 48 h. Colonies (30–300 CFU per plate) were counted, and results were expressed as log CFU/g.

### 2.3 Determination of K value and TVB-N

The K-value of anchovy muscle was determined according to SC/T 3048-2014, 2014, with minor adaptations. The edible dorsal-lateral muscle was dissected and finely minced on ice. A 2.00 g portion of mince was extracted twice with 10% (v/v) HClO₄, each followed by centrifugation at 8000 rpm for 10 min at 4 °C. The combined supernatants were adjusted to pH 6.0–6.4 with NaOH, diluted to 50.0 mL with ultrapure water, re-centrifuged, and filtered through a membrane. Separation was performed on an Agilent ZORBAX Eclipse Plus C18 column (250 mm × 4.6 mm i.d., 5 μm particle size) at 35 °C with a mobile phase of 0.02 mol/L KH₂PO₄–K₂HPO₄ (1:1, v/v, pH 6.0) under isocratic elution at a flow rate of 1.0 mL/min. Detection was at 254 nm with an injection volume of 20 μL. Quantification was carried out using external standards of ATP, ADP, AMP, IMP, HxR, and Hx. The K value was calculated as:

$$K\left(\%\right)=\left(M_{\mathrm{HxR}}+M_{\mathrm{Hx}}\right)/\left(M_{\mathrm{ATP}}+M_{\mathrm{ADP}}+M_{\mathrm{AMP}}+M_{\mathrm{IMP}}+M_{\mathrm{HxR}}+M_{\mathrm{Hx}}\right)\times 100$$

where M denotes the molar concentration (μmol/g).

TVB-N was determined by the Conway micro-diffusion method (Conway & Byrne, 1933). The homogenized anchovy muscle was extracted with 10 g/L trichloroacetic acid (TCA) for 30 min at room temperature. The extract was diluted to 100 mL and filtered. A 1.00 mL aliquot of the filtrate and 1.00 mL of saturated potassium carbonate solution were placed on opposite sides of the outer chamber of a Conway dish. The central chamber contained 1.00 mL of boric acid absorbing solution (20 g/L H₃BO₃ with bromocresol green–methyl red indicator, pH 4.5). The dish was sealed and incubated at 37 °C for 2 h. The absorbed ammonia was titrated with 0.01 mol/L hydrochloric acid until the color changed from blue-green to gray-violet. TVB-N content was calculated as:

$$\mathrm{TVB}-N\left(\mathrm{mg}/100g\right)=\left(V_{1}-V_{2}\right)\times c\times 14/m\times \left(V/V_{0}\right)$$

where V_1_ and V_2_ are the titrant volumes (mL) for sample and blank, m is the sample mass (g), c is the HCl concentration (mol/L), 14 is the molar mass of N (g/mol), V is the aliquot of filtrate taken for diffusion, and V_0_ is the total volume of the TCA extract.

### 2.4 Determination of histamine content

Histamine in anchovy muscle was quantified according to the method of Tunç et al. (2025). The minced muscle was homogenized with 5% TCA and centrifuged. The combined supernatant was made up to 50 mL with 5% TCA. Histamine was extracted from the acid extract by alkaline butanol partitioning, followed by back-extraction into dilute HCl. The purified extract was derivatized with p-nitroaniline diazonium salt in an ice bath and coupled under weak alkaline conditions to form an orange-red azo compound. Absorbance was measured at 490 nm. The histamine content in the sample was calculated as:

$$X\;\left(\mathrm{mg}/100g\right)=\left(C\times V_{1}\times V_{3}\times f\right)/\left(V_{2}\times m\times 1000\right)$$

where X is the histamine content in mg per 100 g of muscle, C is the histamine concentration obtained from the standard curve (μg/mL), V_1_ is the total volume of the crude extract, V_2_ is the volume of crude extract taken for extraction, V_3_ is the volume of the acidic back-extract, *m* is the sample mass (g), and *f* is any additional dilution factor applied before color development. A standard curve (0–10 μg/mL, R^2^ > 0.99) was prepared by derivatizing histamine dihydrochloride standards under identical conditions; f accounts for any additional dilution applied to bring samples within this linear range.

### 2.5 Measurement of endogenous enzyme activity

Cathepsin B and cathepsin B + L activities were determined fluorometrically using *Z*-Arg-Arg-AMC and Z-Phe-Arg-AMC as substrates, respectively, adapted from the method of Qiu et al. (2020). It should be noted that Z-Phe-Arg-AMC can also be hydrolyzed by other cysteine proteases (e.g., cathepsin K and cathepsin S) potentially present in the crude extract. However, given that cathepsin B and L are the predominant lysosomal cysteine proteases in fish muscle and that the assay was conducted at pH 5.5 (near their optimal pH), the potential interference from other proteases is considered minimal. The minced muscle was homogenized in ice-cold extraction buffer (100 mM sodium acetate, 1 mM EDTA, 0.2% Triton X-100, pH 5.5) and centrifuged at 12,000×*g* for 20 min at 4 °C. The supernatant was collected as the crude enzyme extract. Enzyme activity was measured by incubating the extract with substrate at 37 °C for 30 min, and the release of 7-amino-4-methylcoumarin was quantified fluorometrically at excitation 355 nm and emission 460 nm. One unit (U) of cathepsin activity was defined as the amount of enzyme releasing 1 nmol of AMC per minute per milliliter of extract (U/mL).

Catalase (CAT) activity was determined using a commercial assay kit based on the ammonium molybdate colorimetric method (CATM-W48-N; Milbio, Shanghai, China) according to the manufacturer's protocol with minor modifications. The anchovy edible muscle was homogenized in ice-cold extraction buffer (100 mM sodium acetate, 1 mM EDTA, 0.2% Triton X-100, pH 5.5) and centrifuged at 8000×*g* for 10 min at 4 °C. The supernatant was collected as the crude enzyme extract. CAT activity was measured at 405 nm using a microplate reader following the kit's reaction procedure. One unit (U) of CAT activity was defined as the amount of enzyme catalyzing the degradation of 1 μmol H₂O₂ per minute per gram of tissue (U/g).

### 2.6 Shear force and WHC measurement

Shear force of whole anchovies was determined using a TA.XT Express texture analyzer (Stable Micro Systems, Godalming, UK) equipped with a Warner–Bratzler shear blade (HDP/BSW), following the protocol of Agulheiro-Santos et al. (2025). The fish were sampled from the refrigerated batch stored at 4 °C with periodic ice replenishment. Each fish was briefly rinsed, patted dry, and shear-tested at the mid-abdomen region. Shear force was recorded as the maximum peak force during cutting.

Water-holding capacity of anchovy muscle was measured by a centrifugation method. The minced muscle (5 g) was accurately weighed into a pre-weighed centrifuge tube and centrifuged at 5000×*g* for 15 min at 4 °C. After centrifugation, the supernatant was decanted, and the remaining pellet was weighed. WHC was expressed as the percentage of retained water relative to the initial sample weight. The WHC was calculated as the percentage of retained water relative to the initial moisture content, using the following formula:

$$\mathrm{WHC}\left(\%\right)=W_{2}/W_{1}\times 100\%$$

where W_1_ is the sample weight before centrifugation and W_2_ is the sample weight after centrifugation.

### 2.7 Histological and ultrastructural analysis

Histological processing of anchovy muscle followed the workflow described by Díaz et al. (2003) for anchovy tissues. Briefly, whole fish were decalcified in 10% EDTA (pH 7.4) at 37 °C for two weeks, then fixed in 10% neutral buffered formalin. After dehydration and paraffin embedding, 5 μm serial sections were cut using a rotary microtome. Sections were mounted on poly-l-lysine-coated slides, deparaffinized, rehydrated, and stained with hematoxylin and eosin (H&E). Stained sections were dehydrated, cleared, coverslipped, and examined under a light microscope (Olympus BX53, Japan) to observe the histological architecture of muscle and connective tissues.

Ultrastructural observation of anchovy muscle followed the fish muscle TEM workflow of Tan et al. (2024). Mid-lower abdominal muscle blocks were fixed in 2.5% glutaraldehyde in 0.1 M phosphate buffer (pH 7.0) at 4 °C for 24 h. After buffer rinses, tissues were post-fixed in 1% osmium tetroxide, then dehydrated through a graded ethanol series and embedded in resin. Ultrathin sections were cut using an ultramicrotome, stained with uranyl acetate and lead citrate, and observed under a transmission electron microscope.

### 2.8 Proteomic analysis by Orbitrap Astral DIA

#### 2.8.1 Experimental design and sampling

For comparative proteomics, approximately 150 g of decapitated and eviscerated anchovy muscle from each storage batch was frozen in liquid nitrogen and pulverized using a laboratory grinder. The resulting frozen powder was used for protein extraction. Three independent biological replicates were prepared for each time point (*n* = 3 per group), each replicate consisting of an independent 150 g portion taken from the same storage batch. The samples were labeled A1–A3 (day 1) and B1–B3 (day 7). No pooling across time points or replicates was performed. All frozen powders were stored at −80 °C until protein extraction.

#### 2.8.2 Protein extraction and digestion

Frozen anchovy powder prepared as described above was directly lysed in 8 M urea lysis buffer. After centrifugation, the supernatant was collected, and protein concentration was determined using a BCA assay. For digestion, 100 μg of protein was reduced, alkylated, and digested overnight at 37 °C with trypsin at an enzyme-to-protein ratio of 1:50 (w/w). The resulting peptides were desalted on C_18_ SPE columns and quantified.

#### 2.8.3 LC–MS/MS acquisition (Astral DIA)

Peptides were separated on a Vanquish Neo UHPLC system coupled to an Orbitrap Astral mass spectrometer (Thermo Fisher Scientific) using a PepMap Neo column (150 μm × 15 cm, 2 μm) at 55 °C. A 200 ng peptide load was separated over an 8 min gradient at 2.5 μL/min with 0.1% formic acid in water and acetonitrile. DIA acquisition covered 380–980 *m*/*z* at 240,000 resolution, with 299 variable windows (2 Th isolation, 25% HCD, 500% AGC target).

#### 2.8.4 Bioinformatics analysis

The MS/MS spectra were searched against the UniProt zebrafish (*Danio rerio*) protein database (UP000000437, version 2024_01) using Proteome Discoverer 2.5 (Thermo Fisher Scientific) with the Sequest HT search engine. Search parameters were as follows: trypsin digestion with a maximum of two missed cleavages; carbamidomethylation of cysteine as a fixed modification; oxidation of methionine and acetylation of the protein N-terminus as dynamic modifications; precursor mass tolerance of 10 ppm; fragment mass tolerance of 0.02 Da. The FDR was controlled at 1% at both the peptide and protein levels using the target-decoy strategy. Only proteins with at least two unique peptides were considered for further analysis. Differentially abundant proteins were defined by |fold change| > 1.5 and *p* < 0.01. KEGG enrichment was performed with clusterProfiler (Benjamini–Hochberg *p* < 0.05). GSEA was conducted using pre-ranked log₂ fold change against KEGG gene sets (|NES| ≥ 1.5, nominal *p* < 0.05, FDR q < 0.25). PPI networks were built via STRING (confidence ≥0.7) using zebrafish (*Danio rerio*) as the reference organism and analyzed in Cytoscape with MCODE default parameters.

### 2.9 Statistical analysis

Physicochemical, enzymatic, and histological measurements were performed with at least three replicates, and results were expressed as mean ± standard deviation (SD). Statistical differences were evaluated using *t*-test or one-way ANOVA, with *p* < 0.05 considered statistically significant. For proteomic analysis, three independent biological replicates per time point were used (*n* = 3), and differentially abundant proteins between day 1 and day 7 were identified using two-tailed Student's *t*-test with criteria of |fold change| > 1.5 and *p* < 0.01.

## 3 Result and discussion

### 3.1 Sensory and appearance changes during chilled storage

Due to the high protein content and active endogenous enzymes, anchovies exhibited a continuous and obvious visual deterioration during the 7-day chilled storage, which could be divided into four stages: rigor mortis, post-rigor resolution, autolysis, and active spoilage, according to the report of the Food and Agriculture Organization of the United Nations (FAO) (Huss, 1995). As shown in Fig. 1, anchovies on day 1 were in the stage of rigor mortis, which maintained the highest freshness quality. Meanwhile, the fish exhibited highly translucent, off-white muscle with localized blood exudation around the belly, as well as remarkable structural rigidity showing no deformation upon handling. As storage progressed into the post-rigor stage (day 2 and day 3), a subtle shift in muscle color toward a pale yellow tint and the accumulation of lipid exudates on the skin surface were observed. And the firmness of the fish body began to diminish. Subsequently, during the autolysis stage (day 4 and day 5), the fish turned a distinct yellow, and muscle rigidity was completely lost. Despite maintaining overall external integrity, the susceptibility of the flesh to separate from the backbone increased. As lipid oxidation and enzymatic degradation intensified, extensive lipid and sticky mucus appeared. By the final storage phase (day 6 and day 7), the fish progressed into active spoilage with severe structural degradation. The fish exhibited eroded muscle tissue, extensive scale loss, and a thick coating of viscous mucus on the surface. An intense volatile amine off-odor was smelled.

**Fig. 1:**
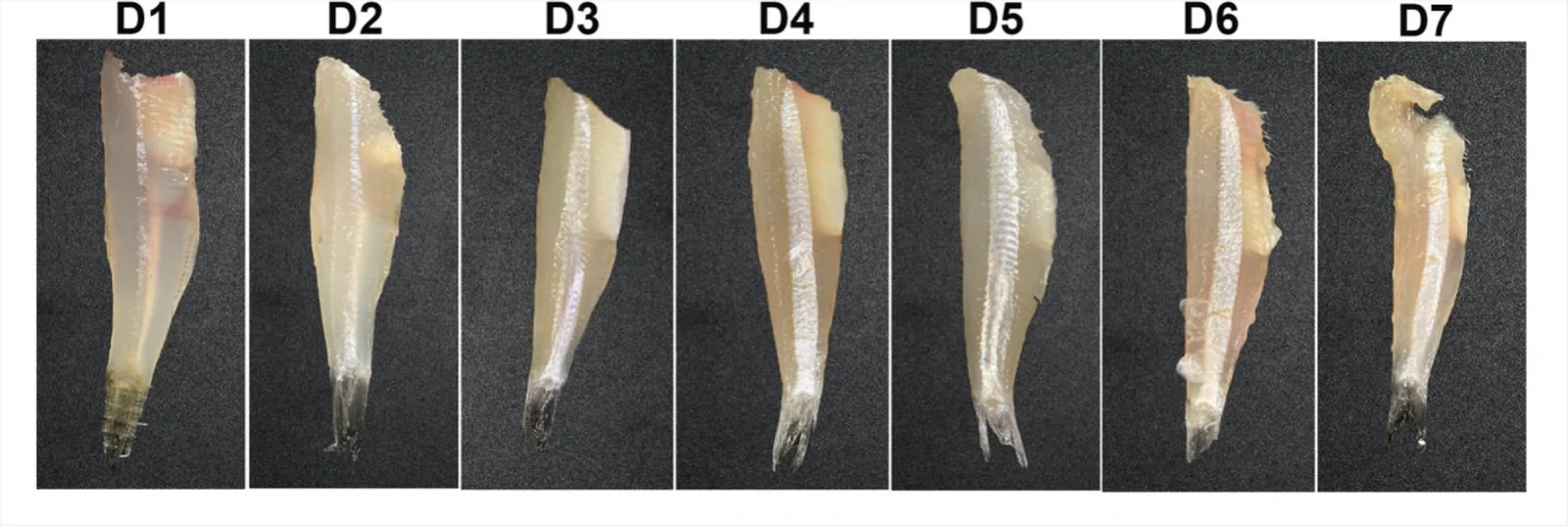
Changes in the sensory appearance of chilled anchovies during 7 days of iced storage.

After death, anchovy muscles remained in a relaxed state for several hours. Then the muscle began to contract, and the whole body became inflexible because of the ATP depletion and Ca^2+^ release. This condition is known as the rigor mortis stage, which has significant technological importance for seafood processing (Gregory & Grandin, 1998). It is best to process fish before the onset of rigor mortis, as muscle contractions could facilitate the blood out thereby improving the whiteness of the fish flesh (Botta et al., 1986). However, as anchovies quickly enter rigor mortis after harvest, the head and viscera removal processes were conducted during this period, resulting in residual blood retention (Fig. 1, D1) and potential microbial contamination. Precision filleting or deboning should be avoided during rigor mortis, because the filleting yield is substantially reduced (Huss, 1995). Therefore, the post-rigor stage (days 2–3) marks the golden window for high-value deboning and filleting for anchovies, as relaxed muscle tension facilitates high yield while maintaining excellent visual quality and strict food safety compliance. A strict rejection criterion for high-value procurement should be applied after the autolysis stage (days 4–7).

### 3.2 Changes of quality traits during chilled storage

The dynamic changes in muscle pH and total plate count (TPC) of anchovies during the 7-day storage were shown in Fig. 2A. The pH value of anchovies decreased slightly from 6.87 to 6.78 between day 1 and day 3, which could be attributed to the accumulation of lactic acid resulting from anaerobic glycolysis (Daskalova, 2019; Yao & Xie, 2026). Subsequently, a rebound in pH was observed from day 4, culminating in a maximum value of 6.97 on day 7. This increase was attributed to endogenous autolysis and bacterial proteolysis, resulting in the accumulation of alkaline nitrogenous compounds (Zhang et al., 2017). Unlike mammalian meat, which exhibits a pronounced postmortem pH decline, the pH of anchovies decreased to a much lesser extent, as their muscle contains less glycogen (Daskalova, 2019). In addition, differences in pH changes may be influenced by species, muscle type, season, diet, and activity level during harvesting (Ocaño-Higuera et al., 2009). Therefore, the relatively high initial pH and limited pH variation in anchovies may be attributed to the prolonged stress during capture, which resulted in higher energy consumption (Poli et al., 2005).

**Fig. 2:**
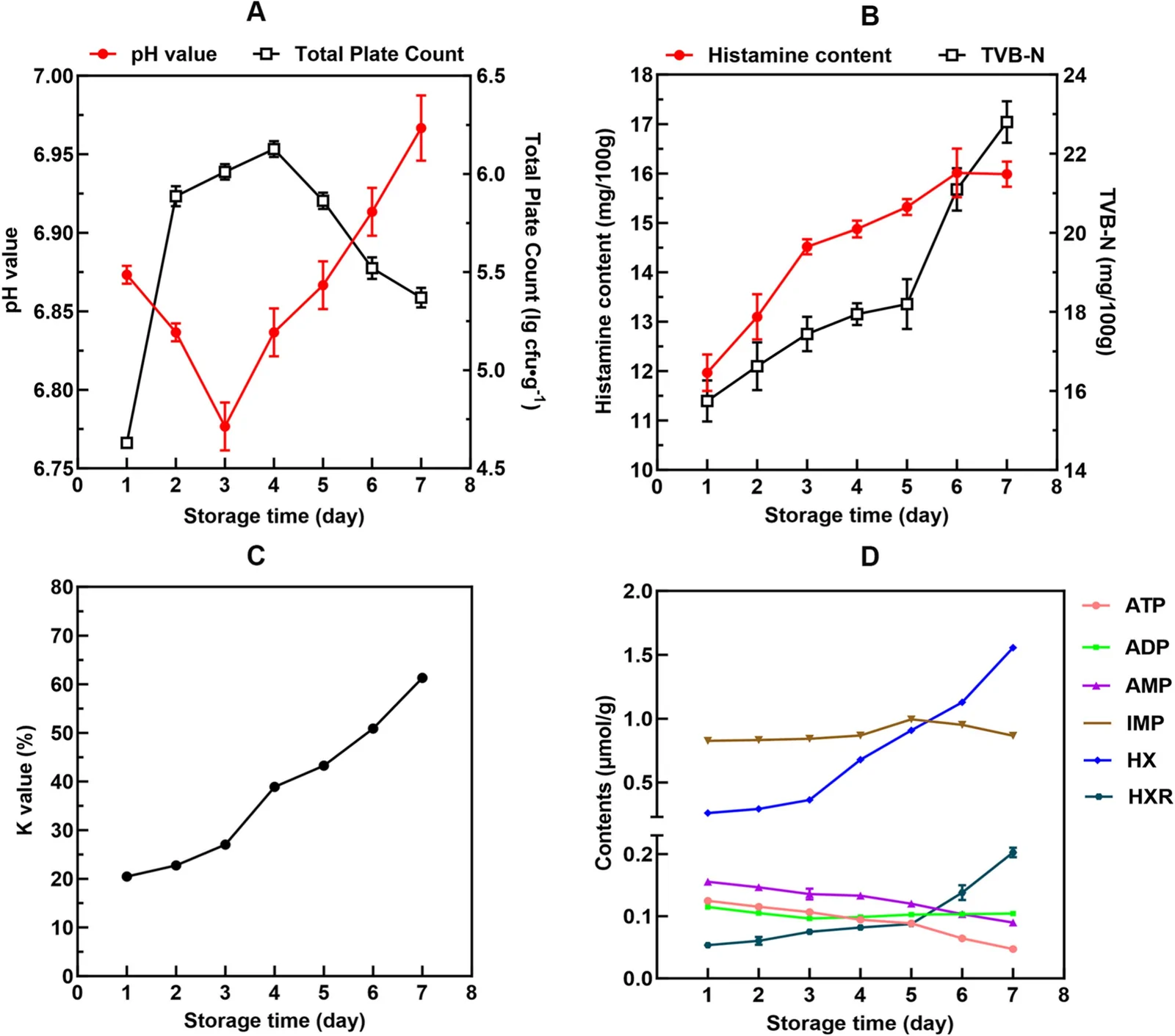
Quality changes of anchovies during 7 days of iced storage. (A) Changes in pH value and Total Plate Count (TPC); (B) Changes in histamine content and total volatile basic nitrogen (TVB-N); (C) Changes in K value; (D) Changes in ATP and its breakdown products (ADP, AMP, IMP, HX, and HXR).

Concurrently, the initial TPC on day 1 was 4.63 log CFU/g and then increased rapidly, reaching approximately 6.15 log CFU/g on day 4. However, the TPC exhibited a continuous decline after day 4, dropping to 5.32 log CFU/g by day 7. This unexpected decrease could be explained by shifts in microbial community structure and limitations associated with conventional aerobic plate counting methods. In order to facilitate transportation and reduce the drip loss, the anchovies were sealed in packaging bags. The oxygen consumption by early colonizers created an increasingly anaerobic microenvironment, and standard aerobic TPC could not support the growth of strictly anaerobic, psychrophilic, and halophilic specific marine organisms during the late storage phase (Parlapani et al., 2014). Additionally, nutrient depletion and the potential production of antimicrobial metabolites by competing microbial populations may also contribute to the observed decline in aerobic plate counts, as reported in vacuum-packaged fish products (Parlapani et al., 2015).

To systematically evaluate the chemical spoilage and safety of chilled anchovies, total volatile basic nitrogen (TVB-N) and histamine contents were monitored over the 7-day storage period (Fig. 2B). Initial TVB-N and histamine values on day 1 were 11.37 ± 0.52 mg/100 g and 11.97 ± 0.37 mg/100 g, respectively, representing fresh raw material status. As storage progressed, TVB-N levels exhibited a moderate increase from day 1 to day 5 (18.19 ± 0.63 mg/100 g), followed by a sharp increase after day 5, reaching 22.80 ± 0.53 mg/100 g on day 7. This sharp rise corresponded to the autolysis stage (day 4 and day 5) and enhanced bacterial activity, which broke down proteins and non-protein nitrogen compounds into volatile amines such as ammonia, trimethylamine, and dimethylamine (Prabhakar et al., 2020). Meanwhile, histamine content increased steadily during the first 3 days to 14.52 ± 0.16 mg/100 g and eventually reached around 16.00 mg/100 g by days 6–7. This trend is mainly attributed to the presence of free histidine in anchovies, which was converted into histamine through microbial histidine decarboxylase-mediated decarboxylation (Oktariani et al., 2022). Throughout the storage, histamine levels remained well below the safety limits established by the US Food and Drug Administration (FDA) (2011) (50 mg/kg) and European Union regulations (100–200 mg/kg) (U.S. Food and Drug Administration, 2022; Commission Regulation (EU) No 1019/2013, 2013), confirming that anchovies are not intrinsically a high-histamine-risk species. According to the official food safety survey and risk assessment report (No. NSW/FA/F1079/1007) published by the NSW Food Authority in 2010, dried anchovies contained an average histamine level of 35 mg/kg, indicating that anchovies are not intrinsically a high-histamine fish species. As for TVB-N, the upper limit for fresh marine fish is 20–30 mg/100 g according to Chinese standard GB 2733-2015, 2015, which indicated that days 5–6 represented a critical chemical turning point. The degradation of adenosine triphosphate (ATP) and the corresponding variation in K value were further investigated. As shown in Fig. 2C, the K-value increased substantially from approximately 20% on day 1 to 61% on day 7, indicating progressive degradation of ATP-related compounds during storage. The K-value exceeded 40% on day 4, which is commonly regarded as the threshold for loss of high-grade freshness in marine fish and aligns with the onset of advanced postmortem deterioration in small pelagic anchovies. As shown in Fig. 2D, ATP, ADP, and AMP were rapidly depleted within the first 5–6 days, while IMP remained relatively stable at 0.8–0.9 μmol/g during Days 1–4 before decreasing gradually. In contrast, Hx and HxR accumulated markedly after Day 4, with Hx reaching 1.5 μmol/g by Day 7. This degradation pattern—i.e., the conversion of IMP (associated with umami flavor) to Hx/HxR (associated with bitter off-flavors)—provides the biochemical basis for the K-value increase and correlates with the gradual loss of umami and development of bitterness during storage, which is consistent with the sensory quality deterioration observed in aged anchovy samples.

### 3.3 Changes in water holding capacity and shear force during storage

The changes in water-holding capacity (WHC) and peak shear force of anchovy muscle during 7 days chilled storage are shown in Fig. 3A. Overall, both WHC and shear force exhibited a continuous downward trend as storage progressed, reflecting progressive textural softening and reduced water-binding capacity of muscle cells. The WHC on day 1 was 88.87 ± 0.22%, indicating an intact myofibrillar network in fresh muscle tissue, and dropped to 82.05 ± 0.31% in seven days, with the steepest decline occurring between day 2 (88.38 ± 0.32%) and day 3 (86.03 ± 0.44%). The most rapid WHC decline occurred during days 2–3, preceding peak cathepsin activity (Day 4), suggesting that early water loss was driven primarily by rigor-induced actomyosin contraction rather than proteolysis, while the later decline reflected proteolysis-mediated structural damage. Concurrently, the peak shear force decreased continuously from an initial value of 3.26 ± 0.14 N on day 1 to 1.95 ± 0.21 N on day 7, indicating a 40.18% reduction in mechanical resistance. The simultaneous decrease in WHC and shear force could be attributed to postmortem enzymatic degradation and structural deterioration of myofibrillar proteins. This loss of structural integrity weakens the constraint on intramyofibrillar water, allowing immobilized water to be released as free water (Xu et al., 2026).

**Fig. 3:**
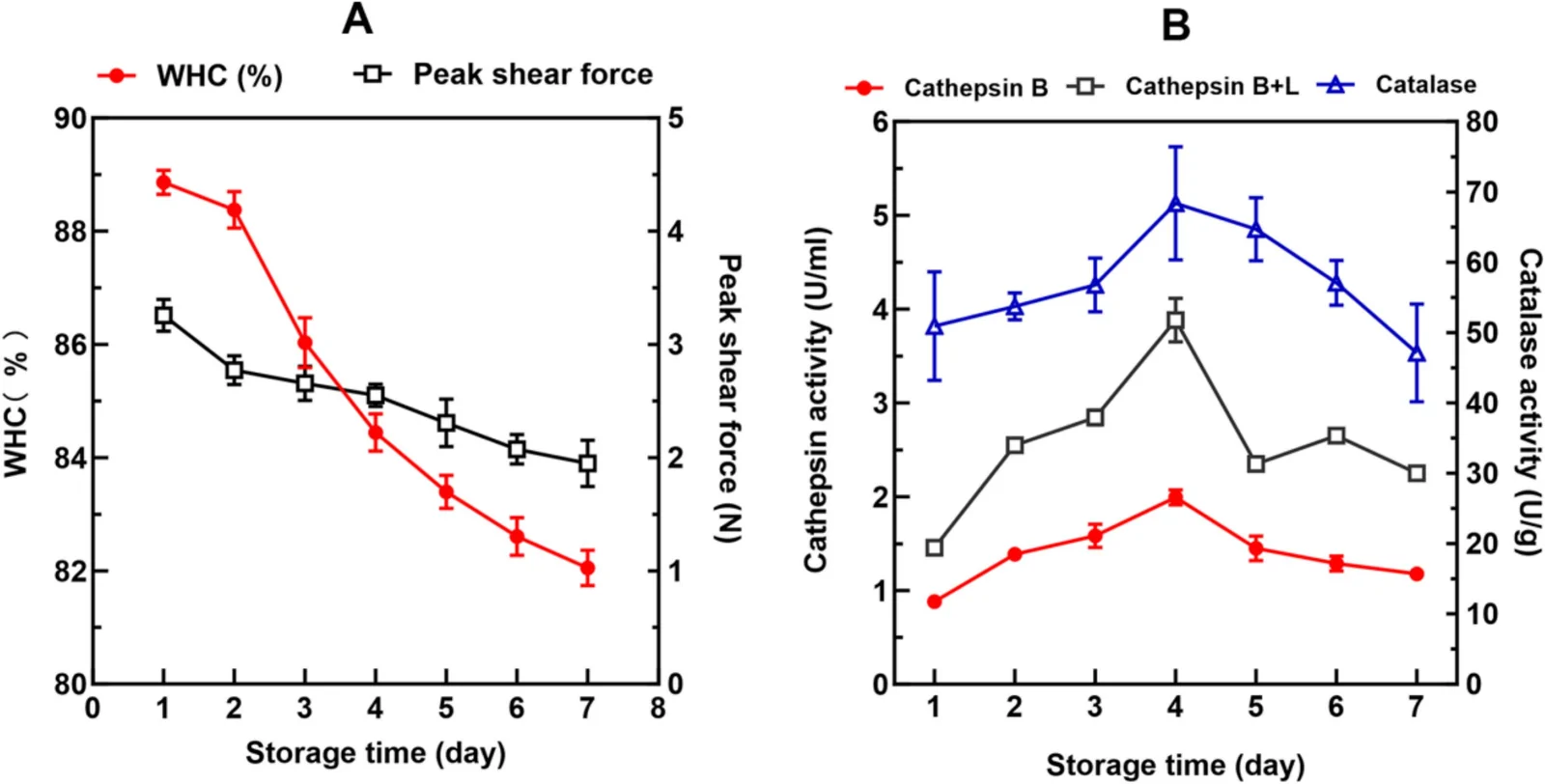
(A) Water holding capacity (WHC) and peak shear force changes of anchovies during 7 days of iced storage. (B) Enzyme activity changes of anchovies during 7 days of iced storage.

### 3.4 Changes in proteases and catalase activities during storage

Myofibrillar proteolysis is closely associated with endogenous protease activity, especially cathepsin B and cathepsin L, which are two key endogenous enzymes involved in postmortem autolysis and muscle texture softening (Jiang et al., 2022). As shown in Fig. 3B, the initial activities of cathepsin B and cathepsin B + L were 0.88 ± 0.04 U/mL and 1.46 ± 0.02 U/mL, respectively. Then, both enzyme activities increased substantially, peaking on day 4 at 1.99 ± 0.08 U/mL and 3.88 ± 0.23 U/mL, respectively. After day 4, cathepsin activities declined to 1.18 ± 0.02 U/mL (cathepsin B) and 2.25 ± 0.03 U/mL (cathepsin B + L) by day 7. Similar trends were observed in yellow croaker and grass carp, and the increase during the early stage could be attributed to the postmortem apoptosis, which facilitated the release of proteases (Fan et al., 2025; Xu et al., 2026). In order to further investigate the change of intracellular oxidative stress level, the activity of catalase (CAT) was measured. As shown in Fig. 3B, CAT activity increased from 50.94 ± 7.74 U/g (day 1) to a maximum of 68.38 ± 8.06 U/g (day 4), before decreasing to 47.11 ± 6.98 U/g at the end of storage. The early upregulation of catalase activity suggested an increased oxidative stress response (Bekhit et al., 2013). After day 4, the decreases of three enzyme activities were associated with enzyme denaturation and reduced stability under chilled conditions (Xu et al., 2024). Overall, day 4 represented a critical biochemical turning point, where maximal enzymatic release accelerates structural collapse and quality failure in chilled anchovies.

### 3.5 Histological changes in anchovy muscle microstructure during chilled storage

The microstructural evolution of anchovy muscle tissue during 7 days chilled storage was investigated via paraffin section microscopy and transmission electron microscopy (TEM). As shown in Fig. 4A and Fig. 4B in the transverse and longitudinal directions, respectively, the fresh muscle tissue on day 1 exhibited an intact and highly organized cellular architecture, characterized by well-defined polygonal muscle fibers separated by distinct extracellular spaces, and longitudinal sections showed parallel aligned fascicles with clear interstitial gaps between adjacent fiber bundles. These ultrastructural changes are consistent with the observed decline in shear force, as *Z*-line and M-line degradation directly disrupts sarcomere integrity and force transmission along the myofibril, resulting in progressive texture softening. Meanwhile, the TEM images (Fig. 5A) showed that myofibrillar ultrastructure remained intact and regular alignment on day 1. The sarcomeres maintained distinct boundaries, with the Z-line appearing as a highly dense, dark band. The M-line was clearly delineated at the center of the H-zone within the A band, and the light-I band regions were well-defined. As storage extended, substantial degradation was observed between day 4 and day 5 (autolysis stage). Light microscopy revealed progressive swelling of muscle fiber and fiber bundles, which was accompanied by a significant narrowing of the intercellular spaces. The M line vanished almost completely on day 5 (Fig. 5C). The Z-line underwent pronounced degradation and fragmentation, appearing blurred and significantly less dense. In addition to the myofibrillar degradation described above, mitochondrial morphological alterations were also observed during the later storage period. At days 5 and 7, mitochondria exhibited noticeable swelling with reduced matrix density, partial cristae disruption, and occasional rupture of the outer mitochondrial membrane.

**Fig. 4:**
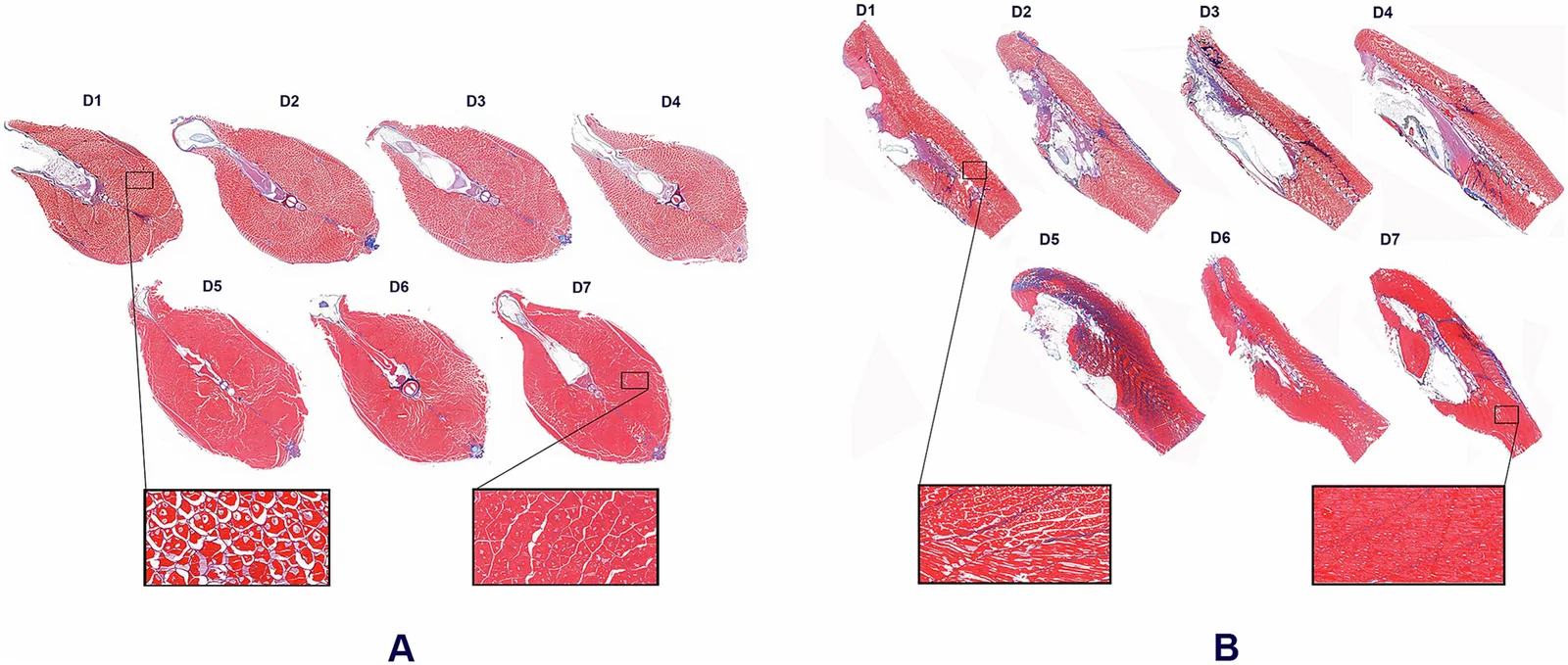
Histological changes in the muscle tissue of anchovies during iced storage evaluated by paraffin sectioning. (A) Transverse sections of anchovy muscle tissue; (B) Longitudinal sections of anchovy muscle tissue.

**Fig. 5:**
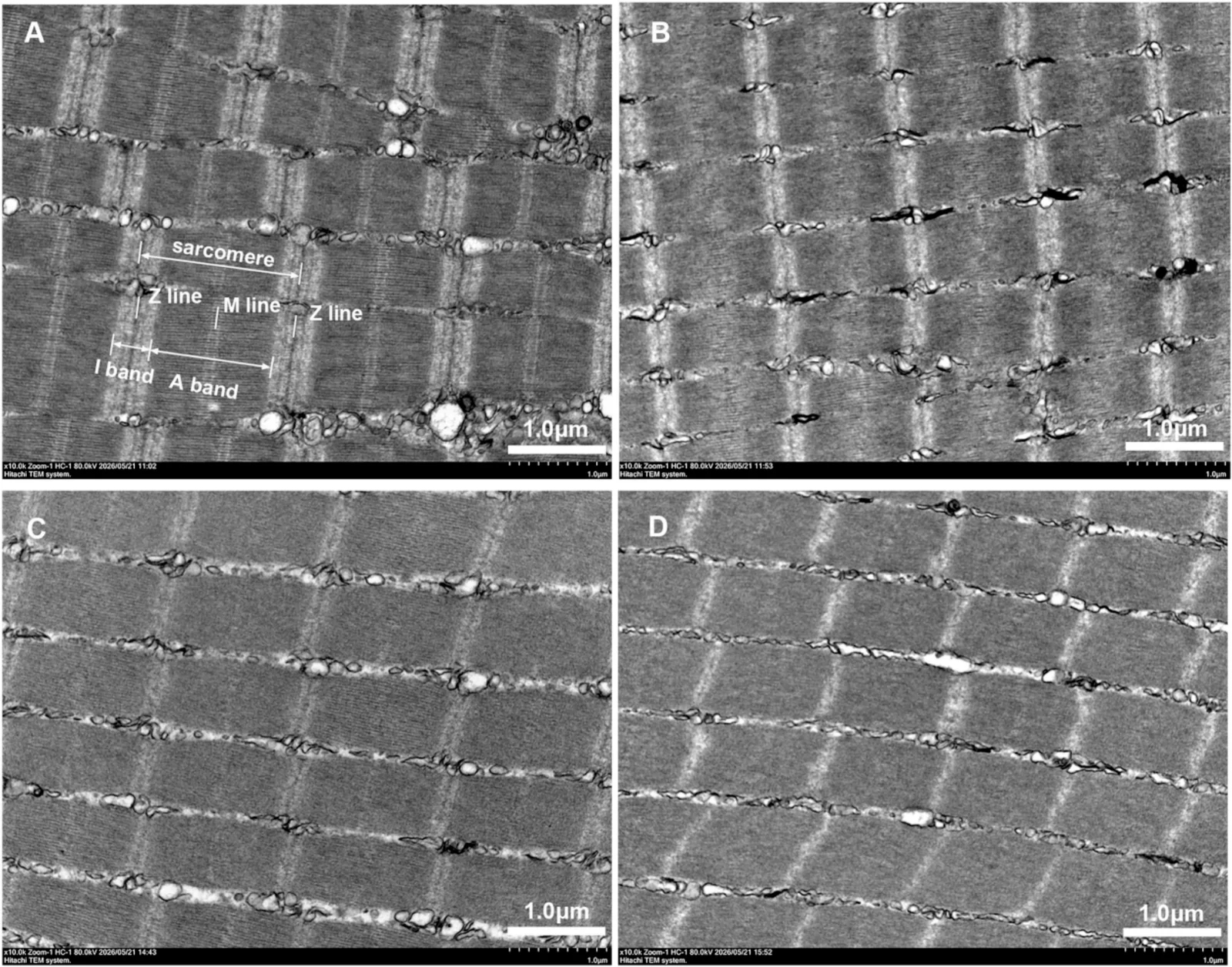
Transmission electron micrographs (TEM) of anchovy muscle tissue during iced storage. (A–D) Micrographs corresponding to days 1, 3, 5, and 7 of storage, respectively (magnification: 10.0 k×; scale bar: 1.0 μm).

Several studies on white-fleshed marine fish species reported a progressive widening of intercellular gaps during storage due to drip loss and myofibril shrinkage (Ayala et al., 2011; Furnesvik et al., 2022). While our results for anchovies showed the opposite trend, which may be attributed to the species-specific characteristics of anchovies as small pelagic fish. During postmortem storage, anchovies rapidly entered rigor mortis, and the depletion of ATP triggered strong actomyosin binding, leading to excessive sarcomere contraction. This intense and non-uniform physical tension induced an inward contraction of the muscle fibers, effectively pulling the muscle fibers away from the endomysial sheath. Consequently, this mechanical pulling generated distinct interstitial gaps and pronounced extracellular cavities visible under light microscopy (Lavety et al., 1988). During the active spoilage stage, the hyper-cleavage of myosin heavy chain (MHC) into smaller peptides drastically increased the intracellular osmotic pressure and exposed numerous hydrophilic amino acid residues (Supplementary Fig. 1). Consequently, this intense postmortem autolysis promoted water absorption and myofibrillar protein swelling due to osmotic imbalance (Xiong, 2005). As storage progressed, the swollen and degraded muscle fibers continuously expanded and pressed against one another, ultimately obliterating the extracellular spaces and fusing into a compacted protein matrix by day 7.

This histological degeneration provided direct microstructural evidence explaining the macroscopic softening texture and reduced WHC of chilled anchovies. The pronounced structural breakdown during days 4–7 was primarily driven by postmortem autolysis and the intense proteolytic degradation of structural and connective tissue proteins by endogenous lysosomal proteases (Jiang et al., 2022). Consequently, the severe histological deterioration observed from day 5 indicated the total collapse of muscle tissue integrity, further corroborating that the optimal raw material quality of chilled anchovies is strictly limited to approximately 3–4 days.

### 3.6 Protein identification

DIA-based proteomics analysis was performed to compare differences in protein abundance between day 1 (D1) and day 7 (D7). A total of 846 proteins and 7110 peptide groups were identified. The identified peptides predominantly ranged from 7 to 20 amino acids in length and were identified based on 1 to 5 peptides mostly, while a small subset of high-abundance or large-molecular-weight proteins exhibited more than 20 matching peptides (Supplementary Fig. 2). These results confirmed the reliability and robustness of the mass spectrometry data for downstream comparative analysis. A total of 257 differentially abundant proteins (DAPs) were identified with filtering conditions of |fold change (FC) | > 1.5 and *p* < 0.01 (Supplementary Table S1). As shown in the volcano plot (Fig. 6A), these screening conditions identified 137 upregulated and 120 downregulated DAPs in D7 compared to D1. Principal Component Analysis (PCA) showed that D1 and D7 proteomic profiles were distinguishable along the first principal component (PC1), which accounted for 55.50% of the total variance (Fig. 6B).

**Fig. 6:**
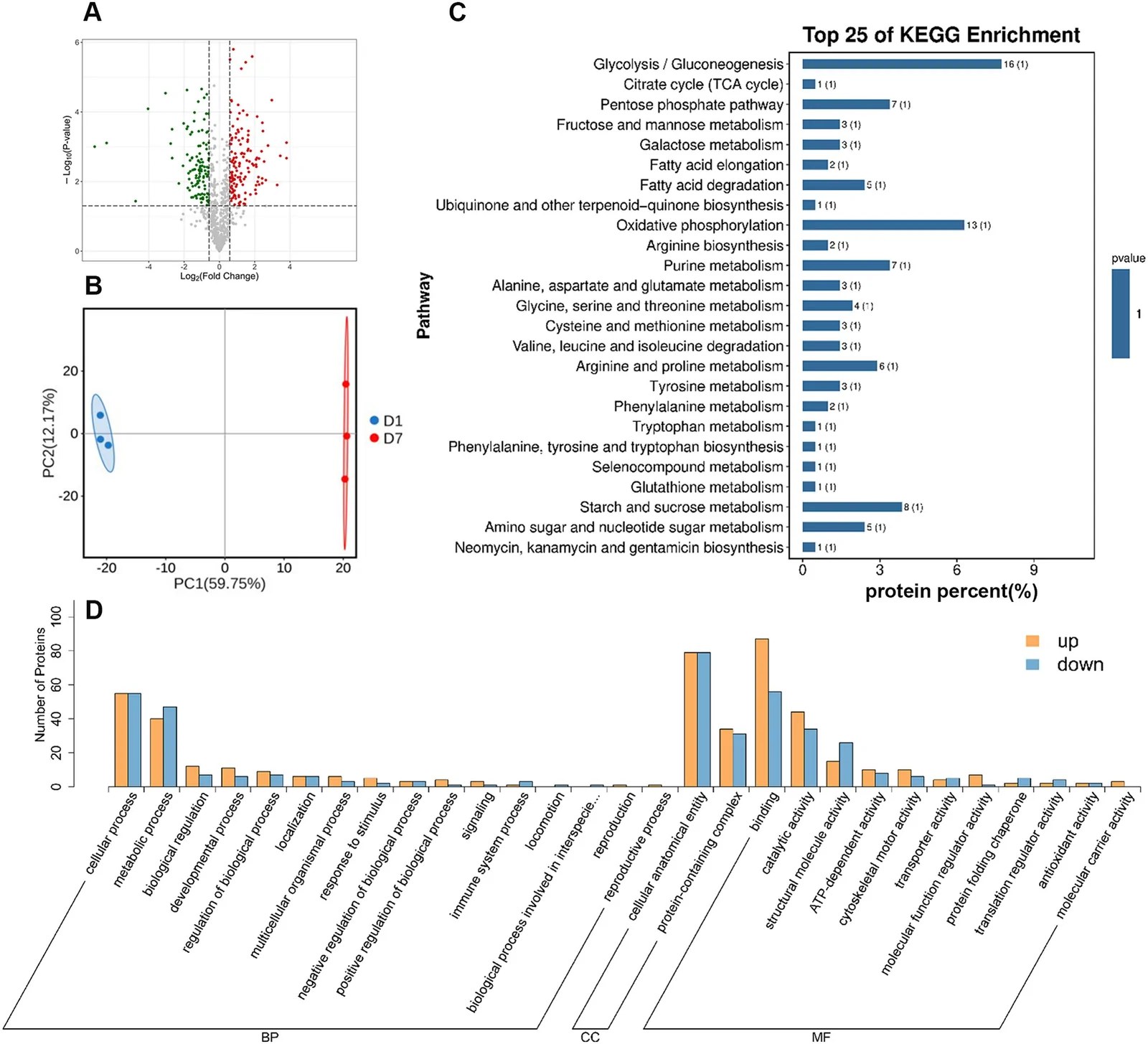
Quantitative and functional enrichment analysis of DAPs in anchovy muscle during iced storage. (A) Volcano plot of DEPs; (B) Principal component analysis (PCA) plot between day 1 (D1) and day7 (D7) groups; (C) Top 25 enriched KEGG pathways for DEPs; (D) GO functional classification with orange and blue bars representing up- and down-regulated proteins, respectively. (For interpretation of the references to color in this figure legend, the reader is referred to the web version of this article.)

### 3.7 Gene Ontology Annotation and KEGG enrichment analysis of DAPs

Gene Ontology (GO) Level 2 classification was performed to analyze DAPs. As shown in Fig. 6D, the DAPs were classified into Biological Process (BP), Cellular Component (CC), and Molecular Function (MF). Orange and blue bars represent up and downregulated proteins, respectively. The most prominently enriched terms were cellular process in BP, cellular anatomical entity in CC, and binding in MF. These functional distribution profiles suggested that postmortem storage induced extensive autolysis, enzyme-mediated proteolysis, and structural disorganization of myofibrillar proteins.

To systematically investigate the biological processes occurring in anchovy muscle during chilled storage, the Kyoto Encyclopedia of Genes and Genomes (KEGG) database was used to map the identified DAPs to functional pathways (Fig. 6C). The top 25 enriched KEGG pathways revealed widespread metabolic alterations, mainly associated with energy metabolism, carbohydrate turnover, and amino acid degradation. Notably, glycolysis/gluconeogenesis was identified as the most significantly enriched pathway, followed by oxidative phosphorylation and starch and sucrose metabolism. This enrichment reflects a metabolic shift toward anaerobic glycolysis associated with postmortem oxygen depletion and ATP consumption. Concurrently, a broad array of amino acid metabolic pathways, such as purine, arginine, and proline metabolism, were also enriched.

### 3.8 GSEA KEGG enrichment of DAPs

Compared with traditional KEGG pathway enrichment, which is sensitive to subjective selection cutoffs and tends to overlook proteins with modest fold changes, Gene Set Enrichment Analysis (GSEA) utilizes the entire dataset to identify coordinated but subtle changes within functional protein sets, which could prevent information loss from hard thresholds and reduce false negatives (Subramanian et al., 2005). As shown in Supplementary Table S2, the D7 vs D1 group contained 52 up-regulated and 19 down-regulated pathways. Among these pathways, two pathways significant enriched in D7, whereas five pathways in D1 were identified and evaluated based on the Normalized Enrichment Score (│NES│ ≥ 1.5), NOM *p* < 0.05, and FDR q < 0.25. Fig. 7A and B showed the two most significant upregulated pathways, including “regulation of actin cytoskeleton” and “vascular smooth muscle contraction.” While the most two significant down-regulated pathways were “ribosome” and “protein processing in endoplasmic reticulum/COVID-19” (Fig. 7C and D). The upregulation of “regulation of actin cytoskeleton” reflects the progressive disassembly of actin filaments and the weakening of the actomyosin network during postmortem storage, directly contributing to muscle softening. This pathway is closely linked to calcium signaling, as actin dynamics are regulated by Ca^2+^-dependent proteins. The enrichment of “vascular smooth muscle contraction” should be interpreted cautiously, as it may partly reflect the presence of vascular smooth muscle cells in the sampled tissue rather than a functional change in skeletal muscle itself. Nevertheless, ATP depletion and calcium release during postmortem storage may also trigger vascular smooth muscle contraction, potentially contributing to microvasculature distortion and blood component release, consistent with the increased hemoglobin subunits observed in the D7 proteomic data.

**Fig. 7:**
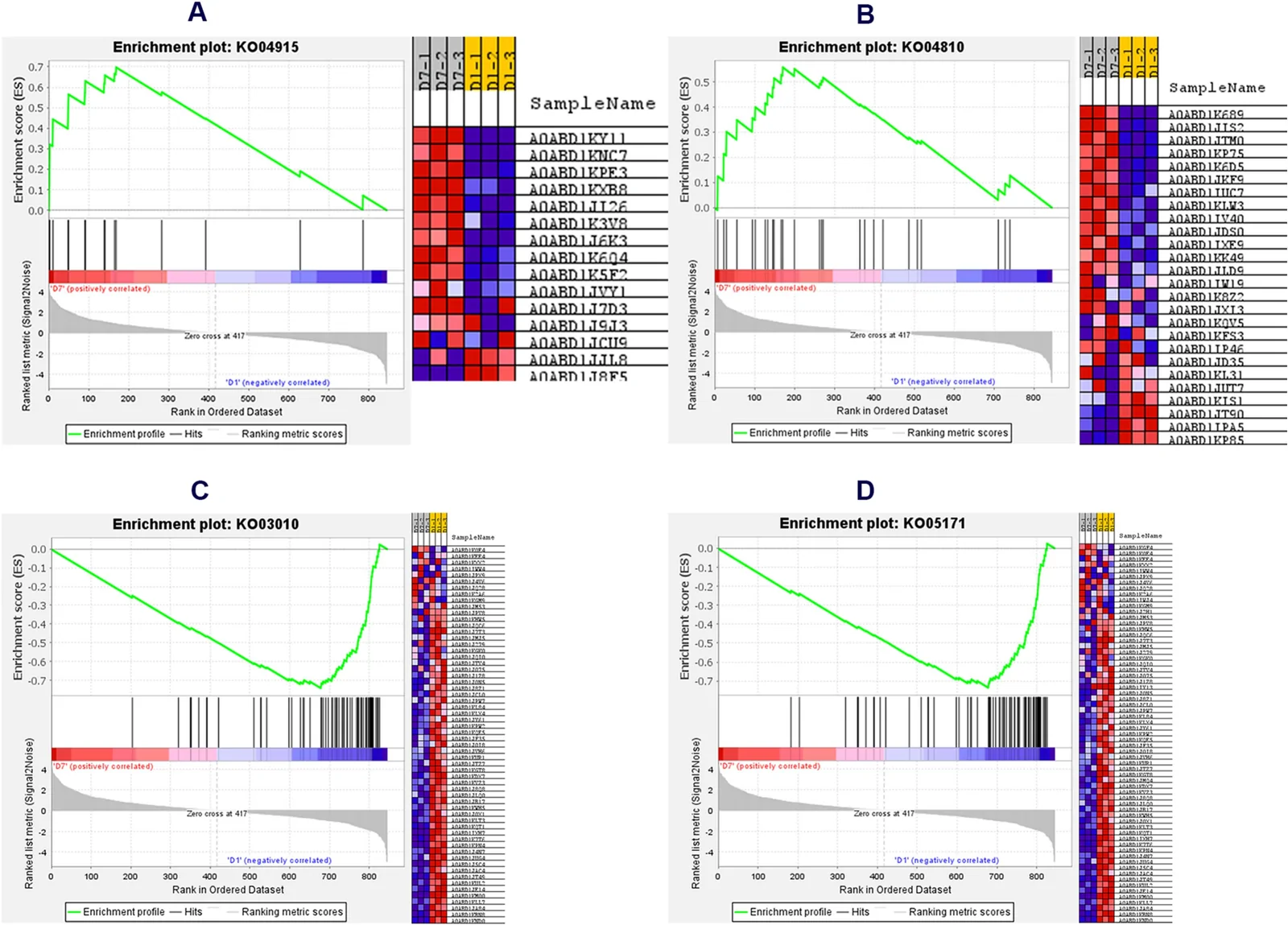
Gene Set Enrichment Analysis (GSEA) of the anchovy muscle proteome during iced storage (D7 vs. D1). (A, B) Top 2 pathways significantly upregulated in the D7 group; (C, D) Top 2 pathways significantly downregulated in the D7 group:

### 3.9 PPI network analysis of DAPs

To investigate the functional organization of DAPs between D1 and D7, protein-protein interaction (PPI) network analysis was performed using the STRING database, and key functional clusters were identified using the MCODE plugin in Cytoscape. Fig. 8A illustrated a cluster associated with muscle fiber structural proteins, which could be further divided into two MCODE-derived modules. Although both modules were predominant in myofibrillar structural proteins, Module 1 comprised the core components of the skeletal muscle contractile apparatus, including fast skeletal myosin heavy chains, skeletal muscle myosin regulatory light chain 2 (MRLC2), and beta-actin. Meanwhile, Module 2 comprised essential light chains (MLC1, MLC4, and myosin light polypeptide 6), non-muscle and smooth muscle myosin variants (myosin-9-like and myosin-11-like), and alpha-cardiac actin. Fig. 8B showed a highly interconnected sub-network comprised of ribosomal proteins, including both 40S small ribosomal subunit proteins (e.g., rps2, rps3a, rps7, rps11, rps15a, rps16, rps19, rps20, and rps21) and 60S large ribosomal subunit proteins (e.g., rpl5, rpl7, rpl10a, rpl12, rpl18a, rpl23, rpl27, rpl30, rpl35, and rplp1). This cluster highlighted extensive alterations in ribosome-associated proteins during storage. Fig. 8C illustrated two clusters associated with energy metabolism. The first module contained key enzymes involved in glycolysis and carbohydrate metabolism. High-degree hub nodes in this cluster included phosphoglucose isomerase, glyceraldehyde-3-phosphate dehydrogenase and fructose-bisphosphate aldolase A/B. The second module comprised mitochondrial inner membrane subunits involved in oxidative phosphorylation, specifically Complex I subunits and cytochrome *c* oxidase subunits.

**Fig. 8:**
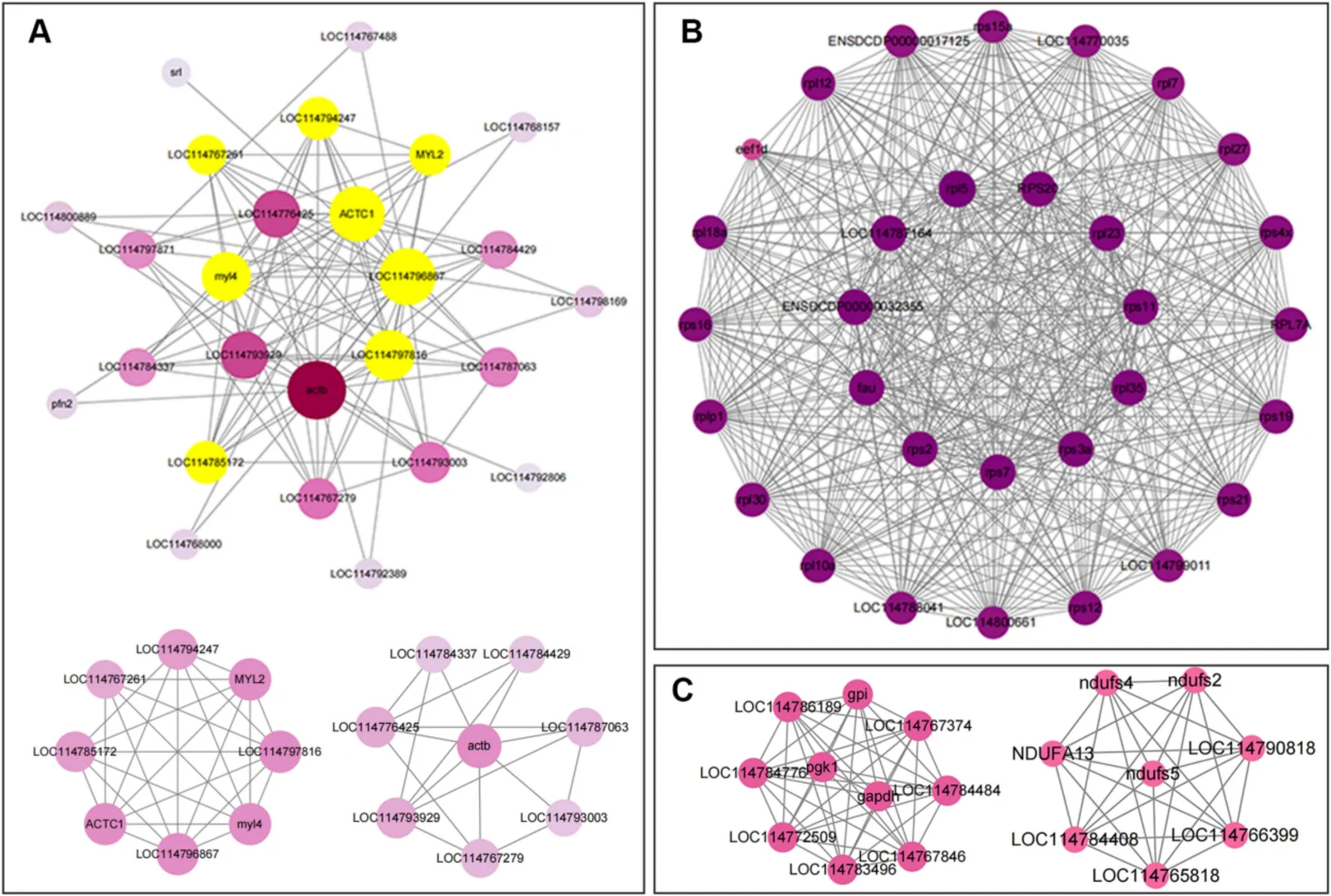
Protein-protein interaction (PPI) network and functional cluster analysis of differentially expressed proteins in anchovy muscle during iced storage. (A) Myofibrillar and cytoskeletal structure proteins; (B) ribosomal proteins; (C) energy/glycolytic metabolism and mitochondrial respiratory chain proteins.

### 3.10 DAPs analysis for potential biomarkers

To systematically investigate the molecular machinery driving postmortem quality failure in chilled anchovies, 51 key functional DAPs were selected based on the criteria (│FC│ > 2.0 and *p* < 0.01), integrated with GSEA enrichment and PPI MCODE network analysis. These core DAPs were classified into muscle structural proteins, mitochondrial apoptosis-related proteins, energy metabolism-related proteins, stress-response chaperones, and calcium regulatory proteins.

#### 3.10.1 Myofibrillar and cytoskeletal structural proteins

As shown in Supplementary Table S3, several intermediate filament (IF) rod domain-containing proteins showed a significant increase in abundance on D7 relative to D1, including type I keratin, type II keratin, and desmin b. These IF proteins constitute one of the three major cytoskeletal networks, extending from the nucleus to the sarcolemma to maintain cell shape and mechanical integrity (Lovering et al., 2011). Structurally, keratins and desmin form a cross-linked filamentous scaffold surrounding the *Z*-disks of sarcomeres and extend peripherally to connect the contractile apparatus directly to the sarcolemma through costamere complexes (Garcia-Pelagio & Bloch, 2021). As keratins are rich in cysteine residues and form more covalent disulfide bonds (Varanko et al., 2020), these proteins were retained within the insoluble myofibrillar-costameric matrix on day 1, rendering them largely unextractable using standard lysis buffers and thus resulting in low mass spectrometry signals. However, as chilled storage progressed to day 7, intense postmortem autolysis disrupted the Z-disk integrity, fragmenting the insoluble keratin-desmin complex into smaller, soluble peptides (Ding et al., 2022). This structural unraveling drastically enhanced the extractability and solubilization efficiency of IF proteins during sample preparation, resulting in an apparent increase in the abundance of type I/II keratins and desmin b in the MS dataset. Therefore, the elevated detectability of these IF rod domain-containing proteins may serve as a potential biomarker of costamere disassembly, Z-disk disintegration, and myofibrillar structural degradation in chilled anchovies.

In contrast, five subunits of the T-complex protein 1 complex were significantly downregulated on day 7. The T-complex protein is a double-ring hetero-oligomeric complex, which assisted the folding and conformational assembly of actin and tubulin (Sternlicht et al., 1993). It has been reported that T-complex protein 1 subunit gamma (P50143) was crucial for maintaining protein structural stability in refrigerated grouper fillets, and its decline directly triggered myoglobin destabilization and significant flesh color deterioration (Zhang & Xie, 2019). This finding was consistent with the increased abundance of hemoglobin subunits observed in our study, which may have contributed to lipid peroxidation and flesh yellowing during the later storage. Additionally, lower abundances of T-complex proteins were also observed in pike eel muscle during chilling and frozen storage, suggesting that these proteins were more prone to denaturation under cold conditions (Yuan et al., 2022).

In addition, members of the myosin superfamily exhibited significant and diverse changes in protein abundance between day 1 and day 7. Specifically, myosin light-chain proteins and regulatory light chains, as well as most myosin isoforms, were enriched in the D7 group. In contrast, myosin heavy chains and myosin motor domain-containing proteins showed lower abundance on day 7. Actin and actin-related proteins such as profilin, plastin-3, and tropomyosin showed increased abundance on day 7. In contrast, F-actin-capping proteins, troponin subunits, and collagen displayed bidirectional abundance patterns. The divergent abundance profiles could be explained by differences in the temporal sequence of postmortem proteolysis and the susceptibility of individual structural domains to degradation (Li, Xie, & Chu, 2023; Li et al., 2026). However, these structural proteins may not be suited as biomarkers for postmortem protein degradation in anchovies.

#### 3.10.2 Mitochondria-dependent apoptosis and energy metabolism

During postmortem storage, a significant increase in the cytosolic abundance of cytochrome *c* oxidase was observed on day 7 (Supplementary Table S4), which was the key apoptotic protein released following mitochondrial damage (Fan et al., 2024). The release of cytochrome *c* oxidase leads to activation of cysteine proteases, especially caspase-3, which cleaves key structural proteins, driving myofibrillar disassembly, *Z*-disk disintegration, and rapid texture softening (Li et al., 2022). Additionally, the apoptosis-inducing factor 1 (AIFM1) and NADH dehydrogenase 1 (Nde1) alpha subcomplex were up-regulated on day 7, and both of them had many obvious similarities in pro-apoptotic signaling with the cell death program (Saladi et al., 2020). AIFM1 is normally localized on the inner mitochondrial membrane, and translocates into the nucleus, where it binds DNA and promotes chromatin condensation and DNA fragmentation which ultimately initiate irreversible cell death upon apoptotic induction (Herrmann & Riemer, 2020). Correspondingly, in our study, a consistent increase in the abundance of histone variants (H1, H3, and H4) was identified in the D7 group. This increase may be associated with AIFM1 translocation to the nucleus and subsequent chromatin condensation during apoptosis. However, histones are nuclear proteins, and their elevated detection in the soluble fraction could equally result from general nuclear lysis during advanced spoilage, which releases nuclear contents into the cytosol. Therefore, the elevated histone abundance likely reflects a combination of AIFM1-mediated nuclear changes and progressive nuclear disintegration, and should not be interpreted solely as evidence of apoptosis.

In the proteomic data, four key glycolytic enzymes, including hexokinase (HK), fructose-bisphosphate aldolase (FBA), phosphoglycerate mutase (PGAM), and pyruvate kinase (PK), were upregulated on day 7. Immediately after fish harvest, blood circulation stopped and oxygen supply ceased. As a result, these glycolytic enzymes may show increased expression as anaerobic glycolysis became the main pathway for generating a small amount of ATP, which was depleted in a few minutes (Daskalova, 2019). Meanwhile, we observed increased abundance of ATPase inhibitor alongside downregulation of adenylate kinase and two ATP synthase subunits on day 7, providing direct evidence for the shutdown of oxidative phosphorylation and the failure of cellular energy homeostasis in chilled anchovies.

#### 3.10.3 Calcium signaling and oxidative stress

During the postmortem period, mitochondrial damage and ATP depletion resulted in increased intracellular Ca^2+^ levels and reactive oxygen species (ROS), which triggered a number of events (Bachs et al., 1992). As shown in Supplementary Table S5, the major calcium-binding protein calsequestrin was decreased in abundance on day 7, which is localized in the sarcoplasmic reticulum (SR) and displayed a high capacity for Ca^2+^ binding (Marabelli et al., 2023). In addition, calcium-transporting ATPase and trimeric intracellular cation channel type A were downregulated on day 7, as well as SR membrane protein reticulon, which plays an essential role in maintaining the membrane curvature and structural network stability (Donahue et al., 2026). Meanwhile, calmodulin-1 was observed to increase in abundance on day 7. These results suggested that the SR experienced severe structural impairment during postmortem storage, and the bound Ca^2+^ with calsequestrin dissociated into the sarcoplasm, where it could no longer be actively pumped back due to the inactivation of SR Ca^2+^ transporters and the disruption of transmembrane charge balance. The rise in sarcoplasmic free Ca^2+^ increased the abundance of calmodulin and rapidly saturated it, subsequently activating calcium-dependent proteases (Buresova et al., 2026). Once activated, calpains vigorously cleaved key sarcomeric structural proteins, including titin, desmin, and myosin, severing the physical linkages between the myofibrils and the sarcolemma.

Meanwhile, ROS accumulation promoted the oxidation of proteins and lipids, as significant enrichment of fatty acid-binding proteins was observed in this study (Ren et al., 2025). Catalase was also up-regulated on day 7, which is one of the key enzymes in antioxidant systems to limit oxidative injury while maintaining signaling functions (Pamplona & Costantini, 2011). According to previous studies, catalase was recognized as a potential biomarker of fish under stress conditions related with oxidation stress (Vasylkiv et al., 2011; Cretu, 2021). In this study, catalase activity peaked on day 4 and then declined, suggesting that it serves as an early-stage indicator of oxidative stress rather than a sustained spoilage marker. This transient pattern reflects the initial antioxidant response to oxidative stress, followed by the progressive loss of enzymatic activity due to protein degradation. Therefore, catalase is proposed as a complementary early-warning biomarker, to be used alongside indicators such as TVB-N and K value that better reflect later-stage quality deterioration. Additionally, heat shock protein 70, a chaperone protein that helps cells fold and repaire proteins during environmental stress, was increased in abundance on day 7. This protein was involved in apoptosis, and could protect muscle structural proteins from degradation by endogenous proteases (Zhao et al., 2025). However, the heat shock protein 70 showed a decreasing tendency in refrigerated carp fillets (Zhang et al., 2025), indicating the difference between among species.

## 4 Conclusion

This study systematically characterized the postmortem quality deterioration of chilled anchovies through an integrated approach combining physicochemical, histological, enzymatic, and DIA-based proteomic analyses. The results demonstrate that postmortem deterioration is driven by a coordinated cascade: mitochondrial dysfunction triggers energy depletion and cytochrome *c* release, activating apoptosis; ATP loss impairs Ca^2+^ homeostasis, causing sarcoplasmic calcium overload that activates calpains and cathepsins; and the resulting proteolytic degradation of myofibrillar and cytoskeletal proteins, together with ROS accumulation, leads to *Z*-line disintegration, texture softening, and reduced water-holding capacity. These interconnected mechanisms converge to produce the observed quality decline, with days 4–5 as the critical turning point. Key proteins identified in the D7 versus D1 comparison as potential freshness biomarkers include type I keratin, hemoglobin subunit, heat shock protein 70, histone H1, fatty acid-binding protein, and cytochrome *c* oxidase. Under the constant 4 °C isothermal condition employed here, a 3-day optimal freshness window is recommended for high-value raw material processing. However, this recommendation is specific to controlled isothermal storage; temperature fluctuations commonly encountered in real cold-chain distribution may accelerate or decelerate the deterioration process, and thus the window should be adjusted according to actual temperature profiles in industrial practice. These findings provide a scientific basis for evaluating post-harvest freshness and guiding downstream processing of anchovies.

The following are the supplementary data related to this article.

**Supplementary Fig. S1:**
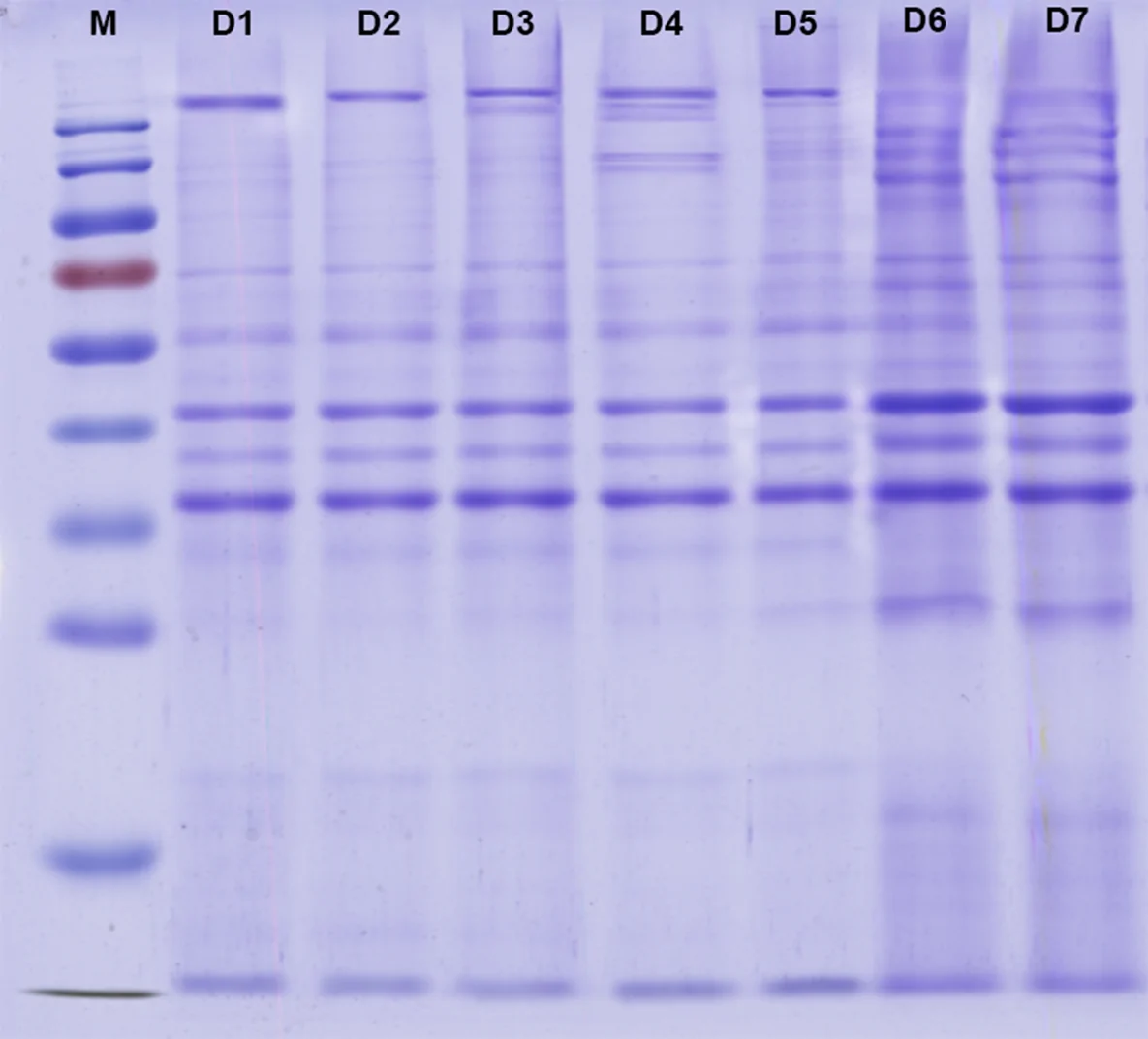
SDS-PAGE electrophoresis of anchovy proteins during iced storage.

**Supplementary Fig. S2:**
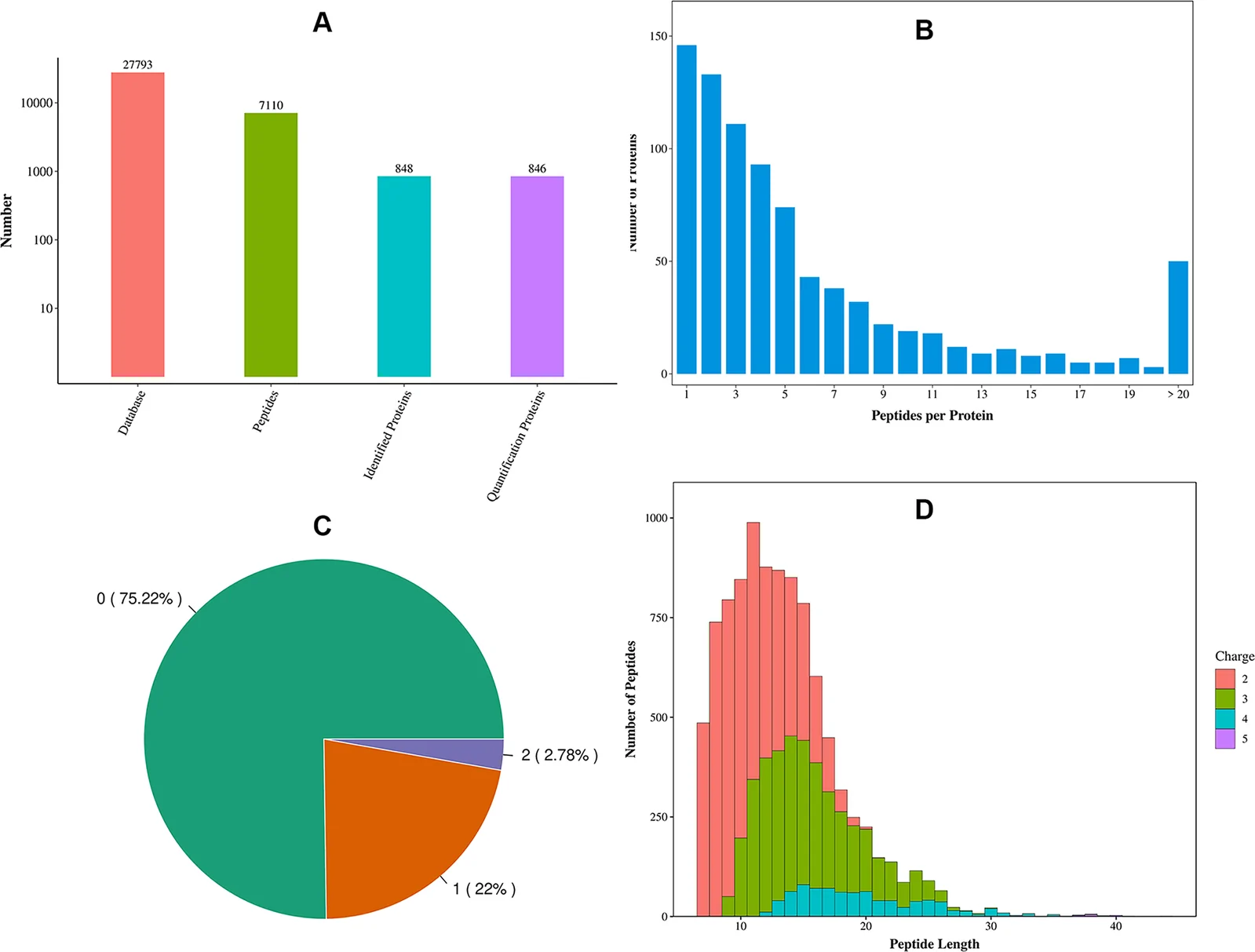
Quality control and quantitative statistics of the proteomic identification.

Supplementary Table S1 The structure, properties, and film-forming advantages of PPs.

Supplementary Table S2 The preparation methods and modification approaches of PPBFs.

Supplementary Table S3 The characteristics of PPBFs.

## CRediT authorship contribution statement

**Shihao Chen:** Writing – original draft. **Jingfu Bai:** Data curation. **Shixian Yin:** Resources. **Wenli Kang:** Funding acquisition. **Hu Hou:** Project administration. **Xiayin Ma:** Writing – review & editing, Writing – original draft.

## Declaration of competing interest

The authors declare the following financial interests/personal relationships which may be considered as potential competing interests: Pingjiang Jinzai Food Co., Ltd. provided anchovy raw materials and partial experimental equipment, but was not involved in the study design, data collection, analysis, interpretation, manuscript writing, or submission decision.

## Data Availability

Data will be made available on request.
