# Supplementary material for: DIA-based proteomic insights into quality deterioration and spoilage biomarkers of chilled anchovies (Stolephorus commersonii)
Source: Food Chem X. 2026 Nov 4;40:104730. doi: 10.1016/j.fochx.2026.104730 (PMC13770631; doi:10.1016/j.fochx.2026.104730)
Supplement: Supplementary file 1 [file mmc3.pdf]

Table S1 Differentially abundant proteins between day 1 (D1) and day 7 (D7) in anchovy muscle during chilled storage. A1–A3: three biological replicates collected on day 1; B1–B3: three biological replicates collected on day 7.

Accession	Gene	A1	A2	A3	B1	B2	B3	B_vs_A_FC	B_vs_A_P-value	B_vs_A_FDR	B_vs_A_Regulation
A0ABD1INT1	ACEWY4_027916	2.0232	2.2595	2.1401	3.7578	3.3648	3.2564	1.6160	0.0056	0.0255	up
A0ABD1IPA5	ACEWY4_004414	54.0676	45.9988	52.9727	35.1815	30.5441	32.0320	0.6388	0.0071	0.0298	down
A0ABD1IPB9	ACEWY4_027561	0.4700	0.5274	0.3145	0.1031	0.2023	0.1338	0.3348	0.0287	0.0714	down
A0ABD1IPY3	ACEWY4_027510	40.6691	38.8220	39.0474	26.9158	25.5886	24.6082	0.6505	0.0001	0.0041	down
A0ABD1IR29	ACEWY4_026947	1.5456	1.4260	1.3395	0.7702	0.7636	0.7779	0.5362	0.0077	0.0311	down
A0ABD1IRH3	ACEWY4_027095	0.3759	0.3030	0.2729	0.5408	0.6439	0.5033	1.7736	0.0114	0.0393	up
A0ABD1IS96	ACEWY4_027223	0.8192	0.5968	0.7038	0.5088	0.4583	0.3770	0.6341	0.0357	0.0834	down
A0ABD1IT43	ACEWY4_025849	1.4600	1.4479	1.2278	9.8379	9.2592	8.7536	6.7341	0.0009	0.0103	up
A0ABD1ITE0	ACEWY4_025945	1.4365	1.4312	1.0720	1.9441	2.3420	2.2253	1.6528	0.0071	0.0298	up
A0ABD1ITF9	ACEWY4_003605	5.0551	4.8286	4.4447	13.6201	14.7606	14.0715	2.9628	0.0001	0.0043	up
A0ABD1ITP6	ACEWY4_027100	0.7405	0.8982	0.8922	4.3505	4.0247	3.3592	4.6365	0.0075	0.0306	up
A0ABD1ITZ7	ACEWY4_026034	1.6087	1.4374	1.9384	4.0790	3.3415	3.4493	2.1807	0.0036	0.0204	up
A0ABD1IUC7	ACEWY4_026667	0.4210	0.4565	0.5535	0.7547	0.7919	0.7371	1.5959	0.0101	0.0362	up
A0ABD1IUL2	ACEWY4_026353	251.4667	249.9420	130.6468	5.3855	9.5249	8.6312	0.0372	0.0367	0.0847	down
A0ABD1IV40	ACEWY4_026409	52.8260	55.3251	46.4337	79.3458	84.0161	70.5222	1.5130	0.0076	0.0308	up
A0ABD1IV74	ACEWY4_026219	0.6734	0.4047	0.4813	1.6164	1.6376	1.4546	3.0195	0.0007	0.0099	up
A0ABD1IV88	ACEWY4_027360	6.1264	5.5750	5.6624	2.4795	2.0733	1.9809	0.3763	0.0001	0.0041	down
A0ABD1IVD3	ACEWY4_024681	0.4435	0.5799	0.5502	0.7797	0.8050	0.8026	1.5171	0.0194	0.0558	up
A0ABD1IVR3	ACEWY4_024790	0.2484	0.2683	0.2560	0.1786	0.1732	0.1551	0.6560	0.0008	0.0101	down
A0ABD1IVZ8	ACEWY4_024882	0.8609	0.9429	0.9121	3.4190	4.3371	4.4435	4.4918	0.0101	0.0362	up
A0ABD1IW72	ACEWY4_024967	52.3750	50.8837	48.2953	34.2745	30.9779	30.6723	0.6329	0.0004	0.0071	down
A0ABD1IX33	ACEWY4_025243	2.3525	2.1958	2.2112	6.4094	5.3828	5.1099	2.5005	0.0124	0.0409	up
A0ABD1IXH7	ACEWY4_025401	3.0465	3.0747	2.8303	1.4123	1.4419	1.3525	0.4699	0.0009	0.0103	down

A0ABD1IXL6	ACEWY4_025398	1.2161	1.0881	1.0934	2.1036	2.1475	2.4567	1.9743	0.0049	0.0235	up
A0ABD1IYG6	ACEWY4_023775	145.5909	138.0394	142.9983	218.1262	221.6782	219.3037	1.5449	0.0001	0.0041	up
A0ABD1IYR2	ACEWY4_025134	0.7445	0.7948	0.7531	0.3050	0.5330	0.3593	0.5223	0.0287	0.0714	down
A0ABD1IYY4	ACEWY4_023936	0.0869	0.1689	0.1233	0.3263	0.2848	0.2449	2.2574	0.0089	0.0335	up
A0ABD1IZ16	ACEWY4_025239	0.3972	0.5644	0.4466	0.2704	0.1237	0.1518	0.3877	0.0130	0.0419	down
A0ABD1IZK3	ACEWY4_024170	16.3661	15.8661	16.4669	9.3403	8.4734	8.9867	0.5503	3.91E-005	0.0028	down
A0ABD1IZL1	ACEWY4_024183	0.8580	0.8134	0.7698	0.1836	0.2987	0.2200	0.2877	0.0003	0.0067	down
A0ABD1IZN7	ACEWY4_024201	0.2170	0.2205	0.2175	0.3724	0.4075	0.3807	1.7719	0.0036	0.0204	up
A0ABD1IZP8	ACEWY4_024162	0.4698	0.5980	0.4131	0.3014	0.2638	0.2078	0.5220	0.0319	0.0769	down
A0ABD1IZQ4	ACEWY4_024215	0.4667	0.4730	0.4857	0.8162	0.8182	0.8311	1.7296	1.58E-006	0.0008	up
A0ABD1IZV0	ACEWY4_024222	0.0912	0.2131	0.2059	0.3929	0.3565	0.3464	2.1477	0.0276	0.0698	up
A0ABD1J0E2	ACEWY4_025355	0.4624	0.4656	0.5920	0.2910	0.0678	0.1124	0.3100	0.0178	0.0521	down
A0ABD1J0N1	ACEWY4_023954	1.5422	1.3217	1.7088	3.4166	3.1596	3.0869	2.1133	0.0004	0.0071	up
A0ABD1J0Q6	ACEWY4_022636	38.1158	38.6541	34.8630	25.4648	23.6247	23.7119	0.6522	0.0024	0.0170	down
A0ABD1J0X1	ACEWY4_024044	3.0491	3.5596	2.4410	1.5361	1.2955	1.7329	0.5044	0.0307	0.0750	down
A0ABD1J1U0	ACEWY4_023031	0.1622	0.3716	0.3083	0.8098	0.8741	1.0436	3.2393	0.0027	0.0176	up
A0ABD1J2H4	ACEWY4_024253	0.4496	0.3106	0.3836	1.6041	1.8431	1.9606	4.7281	0.0022	0.0163	up
A0ABD1J2S6	ACEWY4_023346	0.2524	0.2192	0.2775	0.0975	0.1106	0.1112	0.4262	0.0098	0.0355	down
A0ABD1J388	AK2	0.5300	0.5872	0.4852	0.1542	0.1639	0.1587	0.2976	0.0058	0.0263	down
A0ABD1J3U3	ACEWY4_021610	0.8659	0.9060	0.9370	1.3339	1.6537	1.6047	1.6952	0.0206	0.0586	up
A0ABD1J4H5	ACEWY4_021894	0.2823	0.3749	0.2700	0.1309	0.1474	0.1655	0.4786	0.0310	0.0754	down
A0ABD1J4L9	ACEWY4_023424	96.4706	91.8474	93.5690	155.3516	153.1619	145.4587	1.6105	0.0006	0.0092	up
A0ABD1J4M4	ACEWY4_021954	0.1866	0.0925	0.1350	0.4555	0.3611	0.4972	3.1730	0.0053	0.0247	up
A0ABD1J4N7	ACEWY4_021962	0.8770	0.9185	0.8517	0.4641	0.4226	0.3004	0.4484	0.0046	0.0232	down
A0ABD1J529	ACEWY4_023258	0.6621	0.7221	0.5366	2.8027	2.3966	2.9814	4.2589	0.0038	0.0210	up
A0ABD1J574	ACEWY4_022131	10.6974	10.5319	9.2871	33.2475	30.2653	29.2068	3.0384	0.0013	0.0127	up

A0ABD1J5C4	ACEWY4_022202	2.1227	2.1056	1.8190	0.9265	0.8524	0.9483	0.4510	0.0048	0.0235	down
A0ABD1J5C7	ACEWY4_022196	0.1598	0.1770	0.2838	0.8012	0.8995	0.6736	3.8257	0.0034	0.0200	up
A0ABD1J6E9	ACEWY4_022482	1.9480	1.8754	1.9806	0.9330	0.7968	0.6544	0.4108	0.0019	0.0161	down
A0ABD1J6K3	ACEWY4_022267	5.4151	5.3914	5.6343	9.4626	8.6915	11.1073	1.7798	0.0255	0.0665	up
A0ABD1J712	ACEWY4_020749	0.8412	0.7790	0.6775	0.3002	0.4931	0.2379	0.4488	0.0145	0.0450	down
A0ABD1J7C4	ACEWY4_020875	0.8450	0.5785	0.7936	1.9887	1.6645	1.5417	2.3430	0.0058	0.0263	up
A0ABD1J892	ADSS	2.2011	2.3093	2.7529	0.9653	0.7016	0.6282	0.3160	0.0025	0.0170	down
A0ABD1J8C6	ACEWY4_020724	0.1641	0.1144	0.1446	0.0859	0.0579	0.0712	0.5081	0.0226	0.0624	down
A0ABD1J8C8	ACEWY4_021211	1.4599	1.2110	1.6927	0.4181	0.5410	0.5722	0.3509	0.0137	0.0432	down
A0ABD1J8F5	ACEWY4_022066	3.9946	3.9564	3.5859	1.9727	1.8354	1.8791	0.4930	0.0023	0.0168	down
A0ABD1J8Q8	ACEWY4_022234	2.2406	2.1160	1.7572	1.1596	0.9350	1.1462	0.5301	0.0102	0.0362	down
A0ABD1J9K2	ACEWY4_021033	2.5251	2.1178	2.2153	1.4274	1.1488	1.2348	0.5557	0.0038	0.0211	down
A0ABD1J9R1	ACEWY4_019447	12.2689	12.1758	11.3692	19.4154	18.7791	19.5808	1.6132	4.90E-005	0.0028	up
A0ABD1J9U9	ACEWY4_019493	0.6641	1.4740	1.2093	2.2902	1.7976	2.2770	1.9014	0.0309	0.0752	up
A0ABD1J9W1	ACEWY4_021317	2.0945	2.2161	2.0701	0.6113	0.7827	0.4094	0.2826	0.0017	0.0147	down
A0ABD1JA59	ACEWY4_019586	1.1204	1.2159	1.2649	0.7719	0.7516	0.6547	0.6049	0.0012	0.0117	down
A0ABD1JA84	ACEWY4_019565	0.5735	0.4711	0.4359	0.1900	0.2371	0.1647	0.3998	0.0079	0.0314	down
A0ABD1JAC4	ACEWY4_019658	0.8825	0.7594	0.5912	0.3569	0.2480	0.3744	0.4385	0.0233	0.0631	down
A0ABD1JAD0	ACEWY4_019610	0.5448	0.4199	0.4956	0.8952	0.8193	0.7761	1.7056	0.0024	0.0170	up
A0ABD1JAF0	EIF3A	0.0986	0.0685	0.1114	0.2145	0.1656	0.1693	1.9729	0.0123	0.0407	up
A0ABD1JAL4	ACEWY4_021378	4.9539	5.2391	5.3014	8.4167	8.0171	8.2991	1.5962	4.61E-005	0.0028	up
A0ABD1JB17	ACEWY4_019865	1.2155	1.0678	1.1858	0.5908	0.7123	0.4964	0.5187	0.0028	0.0178	down
A0ABD1JB29	ACEWY4_019906	0.5633	0.4180	0.4579	0.6530	0.7846	0.8116	1.5629	0.0150	0.0459	up
A0ABD1JBC5	ACEWY4_019985	0.9660	0.8198	0.8852	1.7731	1.8657	1.8247	2.0455	0.0002	0.0051	up
A0ABD1JBW2	ACEWY4_020183	2.3518	2.4875	2.2201	6.2332	6.6352	5.8325	2.6491	0.0016	0.0141	up
A0ABD1JC51	ACEWY4_020276	0.1658	0.3625	0.5204	2.0008	2.0247	1.7663	5.5227	0.0004	0.0071	up

A0ABD1JC56	ACEWY4_021371	0.4282	0.3201	0.3373	0.7198	0.5159	0.6208	1.7101	0.0291	0.0722	up
A0ABD1JCF8	ACEWY4_019962	0.5996	0.3866	0.3369	1.2887	1.1332	1.4566	2.9313	0.0025	0.0170	up
A0ABD1JCY7	ACEWY4_020134	0.7645	0.7271	0.9390	1.6474	2.3070	1.5794	2.2767	0.0385	0.0874	up
A0ABD1JDI4	ACEWY4_020344	0.2702	0.1702	0.2785	3.2549	3.1839	3.5826	13.9396	0.0008	0.0101	up
A0ABD1JDX1	ACEWY4_018594	7.2098	7.2907	7.2094	12.0602	11.2441	11.5461	1.6053	0.0027	0.0176	up
A0ABD1JDY8	ACEWY4_018707	0.4699	0.3532	0.3884	0.1259	0.1948	0.1026	0.3494	0.0047	0.0233	down
A0ABD1JE14	ACEWY4_018622	1.7824	2.1615	2.1177	1.0078	0.7392	0.9026	0.4371	0.0025	0.0170	down
A0ABD1JE19	ACEWY4_018728	1.2712	1.3522	0.8487	8.2986	5.9977	7.1627	6.1803	0.0091	0.0341	up
A0ABD1JE22	ACEWY4_018735	7.2223	7.1616	7.3757	12.1790	12.1081	12.6316	1.6967	0.0003	0.0067	up
A0ABD1JE24	ACEWY4_018734	45.6625	32.5270	41.4892	29.5737	17.3687	15.7906	0.5242	0.0318	0.0769	down
A0ABD1JEF6	ACEWY4_018855	1.0513	0.9791	1.1557	0.5651	0.5465	0.2954	0.4416	0.0079	0.0314	down
A0ABD1JHC0	ACEWY4_017617	0.2444	0.0732	0.2329	0.8534	1.0684	0.6856	4.7364	0.0123	0.0407	up
A0ABD1JHC4	ACEWY4_017616	22.9864	20.7116	22.2593	80.0233	78.7694	79.8466	3.6181	2.56E-006	0.0008	up
A0ABD1JHE6	ACEWY4_018856	1.2656	1.3231	1.3145	0.6626	0.6714	0.6050	0.4968	2.20E-005	0.0025	down
A0ABD1JHR9	ACEWY4_017781	0.3157	0.2969	0.2255	0.6343	0.7343	0.7148	2.4859	0.0006	0.0088	up
A0ABD1JI26	ACEWY4_017866	0.8623	0.8304	0.7304	2.0120	1.8112	1.9802	2.3950	0.0003	0.0071	up
A0ABD1JIN8	ACEWY4_018097	2.1502	1.9569	2.2464	3.9070	3.2014	2.9967	1.5905	0.0358	0.0836	up
A0ABD1JIR0	ACEWY4_017996	1.4602	1.8357	1.8879	1.0038	0.7713	1.1404	0.5624	0.0131	0.0421	down
A0ABD1JIS2	ACEWY4_018123	0.1763	0.2188	0.1850	0.6847	0.6883	0.5957	3.3937	0.0013	0.0127	up
A0ABD1JIV7	RPSA	2.4187	1.9547	2.1966	0.9844	1.2253	1.0127	0.4905	0.0045	0.0231	down
A0ABD1JJQ8	ACEWY4_017949	0.2561	0.1609	0.3406	0.6658	0.6321	0.6997	2.6371	0.0085	0.0331	up
A0ABD1JJU5	ACEWY4_016187	2.6622	2.3362	2.0036	3.6096	3.8895	3.2304	1.5324	0.0100	0.0360	up
A0ABD1JKA8	ACEWY4_018210	0.5051	0.5073	0.5984	1.1978	1.2112	1.1238	2.1932	0.0001	0.0041	up
A0ABD1JKB2	ACEWY4_016430	2.2756	1.8385	1.6080	3.5077	3.7101	3.4434	1.8632	0.0067	0.0291	up
A0ABD1JKF9	ACEWY4_017507	6.6147	7.0608	6.9289	12.5027	11.8319	10.7243	1.7015	0.0082	0.0319	up
A0ABD1JKT7	ACEWY4_017627	0.2497	0.1942	0.2526	0.0601	0.1088	0.0887	0.3697	0.0045	0.0231	down

A0ABD1JL69	ACEWY4_016690	0.2410	0.3016	0.1926	0.5179	0.8357	0.6655	2.7464	0.0322	0.0771	up
A0ABD1JLQ0	ACEWY4_016895	1.0060	0.7549	0.9398	0.5562	0.2834	0.3252	0.4312	0.0110	0.0387	down
A0ABD1JLY6	ACEWY4_016498	1.9133	1.9719	1.9599	0.7098	0.5179	0.7617	0.3404	0.0021	0.0163	down
A0ABD1JM46	ACEWY4_016278	0.6122	0.6168	0.4926	0.2329	0.1800	0.2854	0.4056	0.0034	0.0199	down
A0ABD1JMA5	ACEWY4_017027	1.4882	1.7050	1.8420	25.2590	23.2278	21.6445	13.9282	0.0021	0.0163	up
A0ABD1JMB3	ACEWY4_016164	0.3433	0.4076	0.3710	0.1942	0.1355	0.1881	0.4615	0.0016	0.0141	down
A0ABD1JMX7	ACEWY4_015338	6.3677	6.2812	6.2199	1.7951	1.4495	1.3912	0.2457	0.0002	0.0060	down
A0ABD1JN65	ACEWY4_015463	0.3141	0.3051	0.2986	0.0854	0.0747	0.0968	0.2799	2.34E-005	0.0025	down
A0ABD1JNB4	ACEWY4_015554	0.8105	0.8746	0.8874	2.8027	3.9276	3.8162	4.0997	0.0173	0.0510	up
A0ABD1JND8	ACEWY4_015488	4.8756	4.4506	4.7071	8.8947	9.2646	8.4466	1.8959	0.0005	0.0088	up
A0ABD1JNR3	ACEWY4_015707	0.6731	0.5622	0.6471	1.1324	1.1003	1.3816	1.9200	0.0140	0.0438	up
A0ABD1JPX6	ACEWY4_015744	17.4733	16.9012	18.5376	26.7752	27.9707	25.0301	1.5077	0.0023	0.0167	up
A0ABD1JRF2	ACEWY4_014142	2.1212	1.5015	1.5524	0.6716	0.8203	0.5944	0.4031	0.0257	0.0666	down
A0ABD1JRM2	ACEWY4_014208	0.1851	0.2417	0.2394	0.1054	0.1537	0.1171	0.5647	0.0164	0.0489	down
A0ABD1JS49	ACEWY4_014388	23.3964	25.4553	24.5220	43.7440	45.2731	40.4204	1.7641	0.0021	0.0163	up
A0ABD1JSS0	ACEWY4_014598	0.5243	0.3625	0.4034	0.7525	0.5895	0.7181	1.5968	0.0209	0.0592	up
A0ABD1JST9	ACEWY4_014637	0.7021	0.7998	0.7021	0.3958	0.3021	0.1904	0.4030	0.0067	0.0291	down
A0ABD1JT49	ACEWY4_014705	1.7605	1.9017	1.9272	0.8993	0.6939	0.8890	0.4441	0.0004	0.0071	down
A0ABD1JT90	ACEWY4_014708	1.9653	2.0585	2.0246	1.4100	1.3448	1.2254	0.6580	0.0016	0.0141	down
A0ABD1JTB2	ACEWY4_014791	0.1577	0.1685	0.0764	0.6490	0.6345	0.4039	4.1910	0.0222	0.0615	up
A0ABD1JTM0	ACEWY4_014923	0.8075	0.5047	0.8109	2.3301	2.0026	2.2428	3.0970	0.0005	0.0079	up
A0ABD1JTM2	ACEWY4_014883	0.7151	0.9066	0.8704	0.2863	0.2810	0.2757	0.3383	0.0111	0.0387	down
A0ABD1JTZ1	ACEWY4_015003	1.9411	1.6794	1.3165	0.3921	0.4057	0.2070	0.2035	0.0113	0.0393	down
A0ABD1JTZ7	ACEWY4_015005	2.7997	2.7913	2.1108	1.5923	1.2903	1.8347	0.6125	0.0281	0.0707	down
A0ABD1JUG4	ACEWY4_012728	0.9545	0.8885	0.9771	0.2736	0.1029	0.4399	0.2895	0.0153	0.0468	down
A0ABD1JUU2	ACEWY4_012863	0.2893	0.3430	0.2579	0.5195	0.4435	0.4454	1.5821	0.0081	0.0319	up

A0ABD1JV85	ACEWY4_013067	3.0373	2.7296	3.2888	6.1434	5.4830	5.8348	1.9282	0.0004	0.0075	up
A0ABD1JV93	ACEWY4_013084	0.3516	0.4179	0.2991	0.6820	1.1305	1.0982	2.7240	0.0446	0.0961	up
A0ABD1JVX9	ACEWY4_013295	1.9426	2.2442	1.9892	0.9304	1.1523	0.3140	0.3881	0.0259	0.0667	down
A0ABD1JW24	ACEWY4_013335	0.4033	0.2327	0.3689	0.7806	0.6905	0.6096	2.0708	0.0076	0.0307	up
A0ABD1JWK2	ACEWY4_013510	14.7220	16.1729	12.4485	6.3981	7.4706	5.9046	0.4562	0.0094	0.0346	down
A0ABD1JWL0	ACEWY4_013514	7.4882	6.8074	7.3185	3.6626	2.4715	2.1147	0.3816	0.0045	0.0231	down
A0ABD1JWP0	ACEWY4_013522	35.1111	38.4188	41.9662	4.1864	14.3643	16.6802	0.3050	0.0085	0.0331	down
A0ABD1JWT4	ACEWY4_013600	0.2527	0.1619	0.2198	0.3117	0.3833	0.3582	1.6599	0.0162	0.0486	up
A0ABD1JX01	ACEWY4_013511	61.0636	69.6273	70.8013	16.3658	16.0060	15.9211	0.2397	0.0035	0.0204	down
A0ABD1JXZ5	ACEWY4_011563	0.3355	0.4029	0.2867	0.6717	0.5981	0.5010	1.7273	0.0184	0.0532	up
A0ABD1JXZ7	ACEWY4_011547	1.2876	1.2619	1.3339	7.4544	8.0632	6.9370	5.7823	0.0027	0.0176	up
A0ABD1JY44	ACEWY4_011561	5.2146	4.5275	4.4947	2.3217	2.1790	2.0810	0.4623	0.0051	0.0239	down
A0ABD1JYY6	ACEWY4_011882	2.1928	1.4520	2.2233	5.6400	4.7001	4.6912	2.5615	0.0020	0.0163	up
A0ABD1JZ19	ACEWY4_011869	0.6264	0.5738	0.5926	0.2382	0.3525	0.2351	0.4606	0.0071	0.0298	down
A0ABD1JZ72	ACEWY4_011986	0.7044	0.5805	0.4553	1.4414	1.1772	1.2266	2.2097	0.0031	0.0190	up
A0ABD1K0E8	ACEWY4_012201	1.1301	1.3408	1.1713	2.1225	1.9942	1.8250	1.6314	0.0027	0.0177	up
A0ABD1K0F7	ACEWY4_012349	3.1272	2.9100	2.8869	1.7712	1.4012	1.6172	0.5367	0.0008	0.0101	down
A0ABD1K1N1	ACEWY4_010340	0.4176	0.4779	0.4389	1.0260	1.0549	0.7773	2.1419	0.0251	0.0656	up
A0ABD1K1P9	ACEWY4_010347	0.1224	0.1901	0.1670	0.5246	0.6626	0.5629	3.6494	0.0031	0.0190	up
A0ABD1K2P0	ACEWY4_010710	195.9828	209.3843	217.8230	50.9919	28.8584	17.3039	0.1559	0.0003	0.0071	down
A0ABD1K2R0	ACEWY4_010709	71.6920	74.0343	70.1556	151.3365	153.2611	146.0563	2.0875	6.26E-005	0.0033	up
A0ABD1K2R7	ACEWY4_010743	2.3804	2.2031	1.8995	0.3927	0.4005	0.2211	0.1565	0.0021	0.0163	down
A0ABD1K352	ACEWY4_010876	20.5178	19.5097	17.0757	11.3387	11.0015	12.8972	0.6171	0.0071	0.0298	down
A0ABD1K353	ACEWY4_010878	0.3788	0.2926	0.3407	0.4908	0.5836	0.5401	1.5952	0.0055	0.0251	up
A0ABD1K3J6	ACEWY4_011031	25.0471	20.3186	28.8440	10.2131	16.9363	7.8885	0.4721	0.0239	0.0634	down
A0ABD1K3V8	ACEWY4_011133	0.3086	0.2095	0.2399	0.4522	0.4247	0.4801	1.7903	0.0086	0.0331	up

A0ABD1K4M9	ACEWY4_011398	0.2537	0.3968	0.3508	0.1498	0.1552	0.1042	0.4086	0.0302	0.0739	down
A0ABD1K4Y0	ACEWY4_008911	0.2044	0.1350	0.1263	0.4422	0.4646	0.5387	3.1041	0.0012	0.0117	up
A0ABD1K4Y8	ACEWY4_011290	0.0592	0.1347	0.0482	0.3503	0.2909	0.3793	4.2157	0.0023	0.0169	up
A0ABD1K580	ACEWY4_011380	4.4549	4.5836	4.5111	2.9136	3.1023	2.5708	0.6337	0.0063	0.0281	down
A0ABD1K5B7	ACEWY4_011410	2.4393	2.7344	2.3144	5.0758	5.2655	5.0695	2.0580	0.0003	0.0071	up
A0ABD1K5F2	ACEWY4_009052	3.2223	2.9979	3.4614	5.1565	4.7040	4.8925	1.5238	0.0008	0.0103	up
A0ABD1K5M1	ACEWY4_009127	32.7016	32.0963	32.2110	59.0869	58.3132	63.2732	1.8624	0.0027	0.0177	up
A0ABD1K5M4	ACEWY4_009164	0.3026	0.3430	0.2729	0.5246	0.6418	0.4945	1.8084	0.0182	0.0530	up
A0ABD1K5M6	ACEWY4_009141	0.2092	0.4113	0.2669	0.5060	0.5363	0.5707	1.8178	0.0462	0.0988	up
A0ABD1K689	ACEWY4_009362	1.4725	1.1896	1.2807	7.3674	7.9534	5.8108	5.3596	0.0112	0.0390	up
A0ABD1K6D5	ACEWY4_009407	0.1444	0.1551	0.1337	0.2403	0.2776	0.2478	1.7676	0.0030	0.0187	up
A0ABD1K6Q4	ACEWY4_008506	11.2859	12.1150	12.6301	18.8356	18.9547	17.5484	1.5359	0.0005	0.0079	up
A0ABD1K6Q6	ACEWY4_009546	4.0186	4.2631	4.0316	2.5136	2.3237	2.5313	0.5984	0.0001	0.0041	down
A0ABD1K6S2	ACEWY4_009561	45.8748	45.7467	44.5957	68.8898	69.6290	68.0551	1.5165	3.10E-006	0.0008	up
A0ABD1K6T1	ACEWY4_009578	0.1187	0.0888	0.1224	0.6823	0.5708	0.5142	5.3571	0.0081	0.0319	up
A0ABD1K6Z6	ACEWY4_009639	24.7410	24.9759	23.8799	38.9593	41.3602	38.4117	1.6133	0.0014	0.0133	up
A0ABD1K704	ACEWY4_009617	1.2559	1.0782	0.9020	0.5478	0.6723	0.7855	0.6197	0.0355	0.0831	down
A0ABD1K7E7	ACEWY4_009786	0.1491	0.1662	0.0828	0.6997	0.6992	0.6826	5.2285	0.0014	0.0130	up
A0ABD1K7F1	ACEWY4_009794	1.5417	1.6825	1.2895	3.3055	3.6999	3.9537	2.4280	0.0015	0.0141	up
A0ABD1K7P1	ACEWY4_009642	0.8161	0.8797	0.8488	0.4316	0.5741	0.4851	0.5859	0.0061	0.0271	down
A0ABD1K7T6	ACEWY4_009938	2.7234	2.4200	2.3233	1.0798	1.2711	1.1548	0.4695	0.0028	0.0179	down
A0ABD1K7U1	ACEWY4_009921	0.8721	1.0266	0.7621	0.6522	0.6068	0.4676	0.6489	0.0347	0.0820	down
A0ABD1K865	ACEWY4_010057	0.2987	0.2819	0.2866	0.4611	0.6061	0.5462	1.8604	0.0260	0.0668	up
A0ABD1K893	ACEWY4_010088	2.2808	2.2087	1.7086	9.1083	8.5559	9.5739	4.3946	0.0001	0.0046	up
A0ABD1K8S1	ACEWY4_010032	1.3419	1.2113	1.2581	0.1974	0.0866	0.1748	0.1204	2.89E-005	0.0026	down
A0ABD1K9I8	ACEWY4_007887	0.4773	0.4634	0.5659	0.7941	0.9883	0.9566	1.8181	0.0087	0.0333	up

A0ABD1K9N2	ACEWY4_007932	0.4874	0.6691	0.6687	0.8564	1.0521	1.0561	1.6243	0.0134	0.0427	up
A0ABD1KAA3	ACEWY4_008238	0.8887	0.8635	0.9293	2.0726	2.1050	2.1595	2.3633	5.70E-006	0.0010	up
A0ABD1KAB2	ACEWY4_008234	18.4771	17.3006	17.2201	8.9085	10.1775	10.1053	0.5508	0.0002	0.0051	down
A0ABD1KBM6	ACEWY4_008671	9.1516	6.7575	6.0485	2.7325	2.0266	3.0200	0.3543	0.0283	0.0710	down
A0ABD1KBN8	ACEWY4_008715	2.7576	3.0622	3.0606	1.0942	1.2356	0.9563	0.3700	0.0002	0.0055	down
A0ABD1KCE2	ACEWY4_005995	0.7587	0.5780	1.0845	2.0012	1.8212	1.8247	2.3323	0.0100	0.0361	up
A0ABD1KCV7	ACEWY4_006238	8.0608	8.4391	7.1568	4.6177	5.5248	5.0155	0.6407	0.0052	0.0245	down
A0ABD1KDJ4	ACEWY4_006370	68.8049	65.8096	62.5091	0.8431	0.7858	0.7221	0.0119	0.0008	0.0101	down
A0ABD1KDX7	ACEWY4_006612	2.2929	2.4676	2.4046	1.1860	1.3173	1.5723	0.5688	0.0049	0.0235	down
A0ABD1KE79	ACEWY4_006662	0.5883	0.6228	0.6452	1.4748	1.4510	1.2350	2.2415	0.0075	0.0306	up
A0ABD1KE86	ACEWY4_006622	0.9239	1.0472	0.6900	2.4279	2.8463	3.7040	3.3738	0.0236	0.0631	up
A0ABD1KEI4	ACEWY4_006724	0.8215	0.8774	0.7113	0.3902	0.3772	0.2856	0.4368	0.0026	0.0175	down
A0ABD1KEJ2	ACEWY4_006819	8.5291	8.7228	8.8261	18.8552	19.1525	17.9991	2.1477	0.0007	0.0092	up
A0ABD1KER8	ACEWY4_006817	5.3642	3.5437	3.5384	13.2393	11.8544	12.8564	3.0491	0.0006	0.0092	up
A0ABD1KFE7	ACEWY4_006968	20.9857	20.4777	22.7125	0.2163	0.1395	0.1224	0.0075	0.0010	0.0107	down
A0ABD1KGT8	RPS3A	3.2675	3.1637	2.9687	2.0809	1.7597	1.7758	0.5975	0.0009	0.0103	down
A0ABD1KHX0	ACEWY4_005295	0.4048	0.5042	0.5723	1.1591	1.2075	1.1338	2.3629	0.0016	0.0141	up
A0ABD1KID6	ACEWY4_005384	2.2685	2.7081	2.3817	6.5652	6.1361	6.0042	2.5421	9.19E-005	0.0041	up
A0ABD1KIH5	ACEWY4_005435	5.7991	8.0432	7.7654	3.9813	3.1529	1.7679	0.4120	0.0117	0.0398	down
A0ABD1KJ64	ACEWY4_005690	1.6336	1.3342	1.7416	1.0357	1.2413	0.8303	0.6598	0.0349	0.0822	down
A0ABD1KKG8	ACEWY4_004064	0.3048	0.3442	0.3202	0.6362	0.6647	0.5805	1.9413	0.0020	0.0163	up
A0ABD1KKR8	ACEWY4_004159	0.1701	0.0977	0.1312	0.2225	0.2826	0.2134	1.8004	0.0243	0.0640	up
A0ABD1KKX3	ACEWY4_004161	42.7618	42.5090	41.1850	71.1355	67.7817	67.2779	1.6306	0.0006	0.0088	up
A0ABD1KKZ0	ACEWY4_004234	0.8740	0.8417	0.9270	0.4520	0.4693	0.3102	0.4660	0.0040	0.0216	down
A0ABD1KKZ9	ACEWY4_004201	0.3325	0.2604	0.3379	0.2258	0.1432	0.2213	0.6342	0.0366	0.0847	down
A0ABD1KLL7	ACEWY4_004483	0.3283	0.4054	0.3459	0.1460	0.0760	0.1477	0.3425	0.0021	0.0163	down

A0ABD1KLT3	ACEWY4_004467	3.7088	3.7840	3.6057	1.4904	1.9234	2.0621	0.4934	0.0050	0.0239	down
A0ABD1KLW3	ACEWY4_004547	3.5716	4.1584	3.6874	6.1963	5.7453	6.1145	1.5815	0.0008	0.0103	up
A0ABD1KLX4	ACEWY4_004515	1.5400	1.4721	1.2233	0.9419	0.8472	0.9858	0.6552	0.0236	0.0631	down
A0ABD1KM00	ACEWY4_004554	1.6782	1.6548	1.7074	0.7237	0.7322	0.7429	0.4363	5.05E-005	0.0028	down
A0ABD1KM43	ACEWY4_004599	0.2116	0.1604	0.1470	0.2830	0.4551	0.3866	2.1667	0.0420	0.0923	up
A0ABD1KM97	AK1	119.0992	118.6369	114.0333	83.8320	76.4746	72.2947	0.6612	0.0022	0.0163	down
A0ABD1KMT9	ACEWY4_004893	137.6983	136.4755	150.2295	288.9820	311.2451	334.3523	2.2021	0.0029	0.0184	up
A0ABD1KMV7	ACEWY4_004892	19.0303	16.0047	13.6345	9.7985	10.4247	6.8091	0.5554	0.0237	0.0631	down
A0ABD1KMX8	ACEWY4_004884	2.4489	2.0273	2.5458	4.1570	4.9882	4.2974	1.9143	0.0040	0.0216	up
A0ABD1KN37	ACEWY4_002332	64.6321	60.8921	55.4524	19.7472	22.3650	16.0264	0.3212	0.0004	0.0078	down
A0ABD1KNC7	ACEWY4_002448	0.1674	0.1144	0.2147	1.3354	1.9261	1.5328	9.6558	0.0126	0.0412	up
A0ABD1KND0	ACEWY4_002370	1.6918	1.5460	1.7956	0.5870	0.4671	0.6573	0.3400	0.0004	0.0071	down
A0ABD1KNW3	ACEWY4_004882	154.3260	152.5139	175.2928	29.7021	21.5115	19.9140	0.1475	0.0008	0.0102	down
A0ABD1KP75	ACEWY4_002693	0.4868	0.4355	0.5389	1.0323	1.1134	1.1956	2.2868	0.0009	0.0103	up
A0ABD1KP85	ACEWY4_002716	723.8424	644.6863	674.8269	415.5326	436.8522	410.0266	0.6178	0.0038	0.0211	down
A0ABD1KPE3	ACEWY4_002444	0.2170	0.1474	0.2621	1.1625	1.0315	1.1862	5.3951	0.0002	0.0060	up
A0ABD1KPK8	ACEWY4_002504	5.3135	4.9564	4.8872	0.1809	0.3985	0.3411	0.0607	8.12E-005	0.0040	down
A0ABD1KPN4	ACEWY4_002881	2.3072	2.4352	2.7233	1.1118	1.1248	1.2069	0.4612	0.0059	0.0267	down
A0ABD1KPT4	ACEWY4_002873	0.1172	0.0746	0.0626	0.4366	0.3438	0.2950	4.2272	0.0128	0.0415	up
A0ABD1KPW2	ACEWY4_002949	1.6823	1.7623	1.5161	0.9576	1.1461	1.1287	0.6516	0.0040	0.0216	down
A0ABD1KPZ1	ACEWY4_002639	2.0337	1.9448	1.5872	1.2662	1.1207	1.0681	0.6208	0.0221	0.0615	down
A0ABD1KQA9	ACEWY4_003115	9.6227	9.3669	10.2287	17.3709	15.9186	15.0771	1.6554	0.0054	0.0247	up
A0ABD1KQE5	ACEWY4_003133	0.6311	0.6004	0.4666	0.3547	0.4132	0.3280	0.6454	0.0392	0.0881	down
A0ABD1KQT1	ACEWY4_003057	1.1675	1.4245	1.2360	0.6499	0.5120	0.6527	0.4740	0.0036	0.0204	down
A0ABD1KQV3	ACEWY4_003294	13.2433	7.7995	6.8336	76.0175	73.7995	67.9369	7.8114	4.58E-005	0.0028	up
A0ABD1KQV4	ACEWY4_003296	4.7614	4.5236	1.4323	7.2701	8.4204	7.7745	2.1895	0.0478	0.1008	up

A0ABD1KR69	ACEWY4_003426	76.4329	70.0825	56.8880	113.5187	117.8795	111.5380	1.6860	0.0094	0.0345	up
A0ABD1KR96	ACEWY4_003453	0.2397	0.2176	0.2246	0.1564	0.1814	0.1143	0.6631	0.0479	0.1009	down
A0ABD1KRP3	ACEWY4_003568	119.3704	115.2730	113.1323	222.2737	221.1727	204.4622	1.8630	0.0016	0.0141	up
A0ABD1KS73	ACEWY4_003374	3.3211	3.4572	3.4973	1.9980	2.0019	2.2444	0.6077	0.0004	0.0071	down
A0ABD1KUL2	ACEWY4_002013	2.1816	2.1775	2.4594	0.9641	1.2068	0.8418	0.4418	0.0010	0.0103	down
A0ABD1KUR1	ACEWY4_002053	0.6057	0.4407	0.4515	0.3490	0.3293	0.2429	0.6149	0.0476	0.1006	down
A0ABD1KV22	ACEWY4_002182	3.6851	3.5547	3.8724	5.9300	6.3948	5.9437	1.6440	0.0006	0.0088	up
A0ABD1KV70	ACEWY4_002231	74.5006	74.4005	71.5987	20.3275	20.5340	20.4409	0.2780	0.0003	0.0071	down
A0ABD1KVF7	ACEWY4_000018	6.0446	6.3874	5.7943	3.1877	2.8656	3.2080	0.5081	0.0003	0.0071	down
A0ABD1KVP5	ACEWY4_000109	0.8431	0.9121	0.9045	0.1643	0.3633	0.3205	0.3189	0.0051	0.0239	down
A0ABD1KVZ3	ACEWY4_000197	3.1932	3.5634	3.3218	1.9804	2.0573	1.3956	0.5391	0.0071	0.0298	down
A0ABD1KW84	ACEWY4_000275	61.9730	59.9201	59.3712	37.4313	37.4601	35.5924	0.6095	3.11E-005	0.0026	down
A0ABD1KWH5	ACEWY4_000391	0.9953	0.7440	0.6702	0.4452	0.3212	0.4208	0.4927	0.0399	0.0894	down
A0ABD1KWM7	ACEWY4_000426	0.2518	0.1089	0.2457	0.6384	0.6519	0.5737	3.0738	0.0041	0.0217	up
A0ABD1KWP1	ACEWY4_000447	0.4461	0.6255	0.6267	0.8908	1.0492	0.7720	1.5969	0.0315	0.0764	up
A0ABD1KXB1	ACEWY4_000667	1.7937	1.7261	1.8569	4.9880	5.0489	5.0508	2.8061	3.74E-006	0.0008	up
A0ABD1KXB8	ACEWY4_000666	0.9285	0.8703	0.5080	2.0913	2.1798	2.1135	2.7676	0.0073	0.0304	up
A0ABD1KXC9	ACEWY4_000668	0.7081	0.6414	0.6971	2.6209	2.8962	3.0914	4.2063	0.0033	0.0196	up
A0ABD1KXD7	ACEWY4_000687	0.1404	0.1960	0.1342	0.4201	0.3541	0.5297	2.7702	0.0212	0.0597	up
A0ABD1KXX4	ACEWY4_000877	0.5964	0.5014	0.4604	0.9161	0.8727	0.7580	1.6344	0.0065	0.0286	up
A0ABD1KY11	ACEWY4_000920	0.4704	0.5886	0.5963	5.5457	6.4412	6.0707	10.9088	0.0018	0.0154	up
A0ABD1KY16	ACEWY4_000779	1.1823	1.5301	1.4140	0.9235	0.7310	1.0154	0.6470	0.0228	0.0626	down
A0ABD1KY56	ACEWY4_000965	0.5823	0.5165	0.3890	1.4821	1.5934	1.5625	3.1175	0.0004	0.0071	up
A0ABD1KY88	ACEWY4_000992	0.0374	0.1644	0.1016	0.2069	0.3445	0.2395	2.6073	0.0435	0.0944	up
A0ABD1KYV2	ACEWY4_001197	0.7420	0.8757	0.9024	0.4978	0.3771	0.4100	0.5099	0.0035	0.0204	down
A0ABD1KZ83	ACEWY4_000881	0.1178	0.2034	0.2059	0.8336	0.8028	0.6600	4.3569	0.0021	0.0163	up
