# Supplementary material for: DIA-based proteomic insights into quality deterioration and spoilage biomarkers of chilled anchovies (Stolephorus commersonii)
Source: Food Chem X. 2026 Nov 4;40:104730. doi: 10.1016/j.fochx.2026.104730 (PMC13770631; doi:10.1016/j.fochx.2026.104730)
Supplement: Supplementary file 2 [file mmc4.pdf]

Table S2 GSEA enrichment analysis results

NAME	SIZE	ES	NES	NOM p-val	FDR q-val	FWER p-val	RANK AT MAX
KO03010	60	-0.74258345	-2.8264184	0	0	0	168
KO05171	65	-0.7348096	-2.746692	0	0	0	168
KO00071	19	-0.5360252	-1.5264792	0.036480688	0.17910421	0.573	255
KO05165	21	-0.4796863	-1.4410266	0.049065422	0.23710549	0.791	17
KO04512	15	-0.5173579	-1.4114134	0.08017817	0.23297147	0.861	17
KO04979	16	-0.4508854	-1.2341144	0.1893617	0.51677066	0.996	273
KO05017	38	-0.34890553	-1.1856266	0.18181819	0.56572706	0.999	302
KO03050	21	-0.39032853	-1.1767396	0.2522936	0.5173226	0.999	315
KO04151	31	-0.33859983	-1.0881305	0.32461873	0.6758272	1	218
KO05203	16	-0.3985207	-1.0665412	0.36247334	0.6686693	1	22
KO04714	49	-0.278698	-0.9886674	0.495	0.8137171	1	16
KO05205	22	-0.32353085	-0.9567624	0.53452116	0.8306241	1	17
KO00620	25	-0.30509803	-0.95655257	0.49318182	0.7673429	1	34
KO04974	17	-0.33712137	-0.9385271	0.5368421	0.7552917	1	17
KO05146	16	-0.3259352	-0.90442693	0.58719647	0.7855406	1	17
KO05415	69	-0.21759614	-0.8369275	0.8137255	0.882148	1	207
KO00310	15	-0.26233456	-0.71290535	0.85683763	1	1	629
KO00020	17	-0.254413	-0.7097374	0.8730853	0.9566077	1	294
KO00280	30	-0.119454846	-0.3776491	1	0.99984413	1	368
