# Supplementary material for: DIA-based proteomic insights into quality deterioration and spoilage biomarkers of chilled anchovies (Stolephorus commersonii)
Source: Food Chem X. 2026 Nov 4;40:104730. doi: 10.1016/j.fochx.2026.104730 (PMC13770631; doi:10.1016/j.fochx.2026.104730)
Supplement: Supplementary file 3 [file mmc5.pdf]

Table S3. Several key DAPs related with cytoskeletal structure

Classification	Accession	Gene	Description	Fold Change
**IF rod domain containing protein**	A0ABD1JDI4	ACEWY4_020344	type I keratin	13.94
	A0ABD1KNC7	ACEWY4_002448	type I keratin	9.66
	A0ABD1K893	ACEWY4_010088	type II keratin	4.39
	A0ABD1JL69	ACEWY4_016690	desmin b	2.75
**Myosin proteins**	A0ABD1K689	ACEWY4_009362	myosin regulatory light chain 10	5.36
	A0ABD1KY56	ACEWY4_000965	myosin light polypeptide 6	3.12
	A0ABD1JIS2	ACEWY4_018123	Myosin-11a	3.39
	A0ABD1JE19	ACEWY4_018728	myosin-7	6.18
	A0ABD1KNW3	ACEWY4_004882	myosin heavy chain	-6.78
	A0ABD1JX01	ACEWY4_013511	myosin heavy chain	-4.17
	A0ABD1JWP0	ACEWY4_013522	Myosin motor domain-containing protein	-3.28
**Other structural proteins**	A0ABD1ITZ7	ACEWY4_026034	Actin	2.18
	A0ABD1JXZ7	ACEWY4_011547	PDZ and LIM domain-containing protein	5.78
	A0ABD1JTM0	ACEWY4_014923	Profilin	3.10
	A0ABD1IRH3	ACEWY4_027095	Plastin-3	1.77
	A0ABD1JCF8	ACEWY4_019962	Collagen alpha-1 chain	2.93
	A0ABD1IUL2	ACEWY4_026353	collagen, type I	-27.03
	A0ABD1JV93	ACEWY4_013084	F-actin-capping protein subunit alpha	2.72
	A0ABD1KVP5	ACEWY4_000109	F-actin-capping protein subunit alpha-1-like	-3.14
**Chaperone protein**	A0ABD1JDY8	ACEWY4_018707	T-complex protein 1 subunit epsilon	-2.86
	A0ABD1JKT7	ACEWY4_017627	T-complex protein 1 subunit delta	-2.70
	A0ABD1IZ16	ACEWY4_025239	T-complex protein 1 subunit eta	-2.58
	A0ABD1JMB3	ACEWY4_016164	T-complex protein 1 subunit beta	-2.17
	A0ABD1J4H5	ACEWY4_021894	T-complex protein 1 subunit alpha	-2.09
**Histone**	A0ABD1JHC0	ACEWY4_017617	Histone H1	4.74
	A0ABD1JHC4	ACEWY4_017616	Histone H4	3.62
	A0ABD1ITF9	ACEWY4_003605	Histone H3	2.96

Table S4. Key DAPs related with mitochondria-dependent apoptosis and energy metabolism

Classification	Accession	Gene	Description	Fold Change
**Mitochondrial apoptosis-related proteins**	A0ABD1JNB4	ACEWY4_015554	Cytochrome c oxidase subunit 5A	4.10
	A0ABD1IV74	ACEWY4_026219	Cytochrome c oxidase subunit	3.02
	A0ABD1K4Y0	ACEWY4_008911	Apoptosis-inducing factor 1, mitochondrial	3.1
**Energy metabolism-Related proteins**	A0ABD1KCE2	ACEWY4_005995	NADH dehydrogenase1 alpha subcomplex	2.33
	A0ABD1JKA8	ACEWY4_018210	hexokinase	2.19
	A0ABD1ITP6	ACEWY4_027100	Fructose-bisphosphate aldolase	4.64
	A0ABD1J7C4	ACEWY4_020875	Phosphoglycerate mutase	2.34
	A0ABD1K2R0	ACEWY4_010709	pyruvate kinase PKM-like	2.09
	A0ABD1KHX0	ACEWY4_005295	ATPase inhibitor	2.36
	A0ABD1KPK8	ACEWY4_002504	ATP synthase subunit f	-16.47
	A0ABD1J388	AK2	Adenylate kinase	-3.36

Table S5. Key DAPs related with calcium signaling and oxidative stress

Classification	Accession	Gene	Description	Fold Change
**Calcium regulation-related proteins**	A0ABD1KDJ4	ACEWY4_006370	Calsequestrin	-83.83
	A0ABD1KY88	ACEWY4_000992	Troponin C, skeletal muscle	2.61
	A0ABD1K3J6	ACEWY4_011031	Troponin I, fast skeletal muscle	-2.12
	A0ABD1K6Q4	ACEWY4_008506	calmodulin-1	1.54
	A0ABD1JTM2	ACEWY4_014883	Trimeric intracellular cation channel type A	-2.96
	A0ABD1K8S1	ACEWY4_010032	Reticulon	-8.31
**Stress-response proteins**	A0ABD1KY11	ACEWY4_000920	Heat shock protein 70	10.91
	A0ABD1JC51	ACEWY4_020276	fatty acid-binding protein 10-A	5.52
	A0ABD1J2H4	ACEWY4_024253	fatty acid-binding protein	4.73
	A0ABD1K4Y8	ACEWY4_011290	Catalase	4.22
	A0ABD1KQV3	ACEWY4_003294	hemoglobin subunit	7.81
	A0ABD1IT43	ACEWY4_025849	hemoglobin beta embryonic-2	6.73
	A0ABD1KQV4	ACEWY4_003296	hemoglobin subunit alpha	2.19
